# Comprehensive analysis of single-cell and bulk RNA sequencing data reveals an EGFR signature for predicting immunotherapy response and prognosis in pan-cancer

**DOI:** 10.3389/fimmu.2025.1604394

**Published:** 2025-06-12

**Authors:** Changchun Ye, Xiaoya Chen, Zilu Chen, Shiyuan Liu, Ranran Kong, Wenhao Lin, Minxia Zhu, Xuejun Sun, Zhengshui Xu

**Affiliations:** ^1^ Department of Thoracic Surgery, The Second Affiliated Hospital of Xi’an Jiaotong University, Xi’an, Shaanxi, China; ^2^ Department of General Surgery, The First Affiliated Hospital of Xi’an Jiaotong University, Xi’an, Shaanxi, China; ^3^ Department of Thoracic Surgery, The First Affiliated Hospital of Wenzhou Medical University, Wenzhou, Zhejiang, China

**Keywords:** immune checkpoint inhibitors (ICIs), epidermal growth factor receptor (EGFR), pan-cancer, scRNA-seq, immunotherapy response

## Abstract

**Introduction:**

Immune checkpoint inhibitors (ICIs) have changed the paradigm of cancer treatment, but their effectiveness in some patients with epidermal growth factor receptor (EGFR) mutations is unsatisfactory. Therefore, it is necessary to develop a new biomarker for combined immunotherapy strategies to maximize the clinical benefits.

**Methods:**

We collected and investigated 34 pan-cancer scRNA-Seq cohorts from The Cancer Genome Atlas (TCGA) and 10 bulk RNA-Seq cohorts utilizing multiple machine learning (ML) algorithms to identify and verify a representative EGFR-related gene signature (EGFR.Sig) as a predictive biomarker for immunotherapy response. Core genes were identified as Hub-EGFR.Sig to predict the prognosis of cancers and to understand the crosstalk between EGFR and the tumor immune microenvironment (TIME).

**Results:**

EGFR.Sig can accurately predict the ICI response with an AUC of 0.77, demonstrating superior predictive performance compared to previously established signatures. Twelve core genes in EGFR.Sig were identified as Hub-EGFR.Sig, of which 4 immune resistance genes were previously verified in different CRISPR cohorts. Notably, the prognosis most related to Hub-EGFR.Sig was bladder cancer, which can be divided into two clusters with different responses to immunotherapy based on Hub-EGFR.Sig.

**Discussion:**

We developed a promising pan-cancer signature based on EGFR-related genes to serve as a biomarker for immunotherapy response and survival outcome prediction. Furthermore, core genes were identified for future targeting, which will pave the way for improving the effect of immunotherapy in the context of combination immunotherapies.

## 1 Introduction

Immunotherapy with immune checkpoint inhibitors (ICIs) has innovatively expanded the field of cancer treatment and conferred substantial clinical benefits to patients over the past decade (1, 2). Since approved for clinical use, ICIs targeting humanized cytotoxic T lymphocyte antigen 4 (CTLA4), programmed cell death 1 (PD-1) or PD-1 ligand 1 (PD-L1) have been used to treat an ever-growing list of malignant tumors, such as melanoma, lung cancers, head and neck squamous cell cancer, bladder cancer (BLCA), and gastro-esophageal cancer (3). However, only a subset of patients respond to ICIs (4). Hence, the advancement of strategies for accurately predicting ICI response and improving understanding of resistance mechanisms to optimize ICI regimens is paramount (2, 5, 6). All of the above highlight the importance of identifying and developing predictive biomarkers for an ICI response.

Epidermal growth factor receptor (EGFR), which is widely distributed on the surface of epithelial cells in various tissues of mammals, is studied as one of the most well-known kinase receptors in the mechanism of tumorigenesis (7). The robust signaling of EGFR in the activation of prosurvival and antiapoptotic pathways can promote tumorigenesis, proliferation and invasiveness (8–10). Consequently, therapies targeting EGFR have advanced precision oncology. There are currently dozens of EGFR-targeting drugs for various tumors according to the updated directory of the FDA (11), such as gefitinib for the first-line treatment of non-small cell lung cancer (NSCLC) (12) and erdafitinib, which was first approved for urothelial carcinoma (UC) (13), and the drug catalogue is in continuous iteration (7). Therefore, EGFR may have the potential to be a powerful predictive biomarker for ICI response.

Currently, the crosstalk between immunotherapy and EGFR has received increasing attention (14, 15). Several clinical trials in NSCLC have suggested that most EGFR-mutated NSCLC shows a poor response to anti-PD-1/PD-L1 treatment (16–18). Initial results indicated that this phenomenon may be related to the interplay between EGFR and the immune environment, such as weakening immunogenicity through low PD-L1 expression, low CD8^+^ tumor-infiltrating lymphocytes, and low tumor mutational burden (TMB); however, the specific mechanism is unclear (7, 16). Therefore, there is an urgent need for relevant research to comprehensively understand the relationship between EGFR and the tumor immunotherapy response in pan-cancer.

Compared with the traditional study of biomarkers based on the average genetic spectrum of many different cell populations in tumor tissue, the advent of single-cell RNA sequencing (scRNA-Seq) makes it possible to dissect gene expression in the single-cell dimension of malignant tumors, which enables us to identify more accurate and higher performance gene signatures as biomarkers (19). In the present study, we revealed the potential relationship between EGFR and immunotherapy response in two scRNA-Seq cohorts of patients treated with ICI therapy. Subsequently, we conducted a comprehensive analysis of 34 pan-cancer scRNA-Seq and 10 bulk RNA-Seq cohorts to identify a representative EGFR-related gene signature (EGFR.Sig). Furthermore, we extensively investigated and verified the predictive effectiveness of EGFR.Sig for immunotherapy response and identified hub genes in EGFR.Sig (Hub-EGFR.Sig) with the help of multiple machine learning (ML) algorithms to explore the relationship between EGFR and cancer prognosis. Finally, the prognosis of BLCA was found to be most significantly associated with Hub-EGFR.Sig and was analyzed in depth. Our findings more convincingly emphasize the potential of EGFR as a promising biomarker for predicting tumor immunotherapy response and prognosis across multiple cancer types.

## 2 Materials and methods

### 2.1 Data download

A total of 36 EGFR-related genes were obtained through the Gene Set Enrichment Analysis database (GSEA, https://www.gsea-msigdb.org/gsea, **Supplementary Table S1**). Gene set variation analysis (GSVA) can transform the expression matrix of genes into gene sets among different samples to evaluate the results of gene set enrichment (20). According to GSVA, the expression scores of EGFR-related gene sets in the datasets could be obtained.

To research the relationship between EGFR and the immunotherapy response of cancer cells, two scRNA−Seq ICI cohorts with definite curative effects were downloaded from the Gene Expression Omnibus (GEO, https://www.ncbi.nlm.nih.gov/geo) database. One was a melanoma cohort (GSE115978, 33 patient samples, 7186 cells) for analysis (21), and the other was an independent basal cell carcinoma (BCC) cohort (GSE123813, 10 patient samples, 43640 cells) for verification (22).

To construct EGFR.Sig in pan-cancer, 34 scRNA-Seq datasets including 345 patients and 663760 cells were collected from the Tumor Immune Single-cell Hub (TISCH, http://tisch.comp-genomics.org/) portal (23). The TISCH database employs multiple integrated methodologies for comprehensive single-cell annotation within the tumor microenvironment. The datasets covered 17 cancer types, namely, BCC, breast cancer (BRCA), cholangiocarcinoma (CHOL), colorectal cancer (CRC), lower grade glioma (LGG), head and neck cancer (HNSC), liver hepatocellular carcinoma (LIHC), medulloblastoma (MB), Merkel cell carcinoma (MCC), multiple myeloma (MM), neuroendocrine tumor (NET), NSCLC, ovarian serous cystadenocarcinoma (OV), pancreatic adenocarcinoma (PAAD), skin cutaneous melanoma (SKCM), stomach adenocarcinoma (STAD), and uveal melanoma (UVM).

To explore the potential association between EGFR.Sig and the immune response, the transcriptome data of a TCGA pan-cancer cohort comprising 30 different cancer types were downloaded from the UCSC XENA (https://xenabrowser.net) data portal (24). Our analysis excluded some cancer types mainly composed of immune cells, including diffuse large B-cell lymphoma (DLBC), acute myeloid leukemia (LAML) and thymoma (THYM) (25). All downloaded sample data met the following criteria: (a) patients had mRNA expression data and clinical data, (b) patients had completed standardized diagnosis and treatment, including surgery, chemotherapy, and radiotherapy, and (c) survival data were available for patients with survival times greater than 30 days.

TMB was retrieved from cBioPortal (https://www.cbioportal.org) (26, 27). The intratumor heterogeneity (ITH) data were from published research by Thorsson et al. (28). These data were used to analyze the correlation between EGFR.Sig and TMB or ITH.

Ten ICI RNA-Seq cohorts with clinical information were systematically collected to construct a prediction model and subsequent survival analysis. These cohorts included 5 SKCM cohorts (Hugo 2016 (29), Liu 2019 (30), Gide 2019 (31), Riaz 2017 (32) and Van Allen 2015 (33, 34)), 2 urothelial carcinoma (UC) cohorts (Mariathasan 2018 (35), Synder 2017 (36)), 1 glioblastoma (GBM) cohort (Zhao 2019 (37)), 1 gastric cancer (GC) cohort (Kim 2018 (38)) and 1 renal cell carcinoma (RCC) cohort (Braun 2020 (39)). All included cohort samples were collected prior to immunotherapy treatment. For the same patient with multiple tissue samples, an earlier sample was selected, and each patient was counted as one case. After using COMPAT to eliminate the batch effect of different cohorts, the Braun 2020 RCC, Mariathasan 2018 UC, Liu 2019 SKCM, Gide 2019 SKCM and Riaz 2017 SKCM datasets were merged into a large dataset (n=772) according to a previously reported method (40). The combined dataset was randomly divided into a training set (80%, n=618) and a validation set (20%, n=154). In addition, Hugo 2016 SKCM, Synder 2017 UC and Zhao 2019 GBM were used as independent verification sets to evaluate the predictive value of the model.

**Figure 1:**
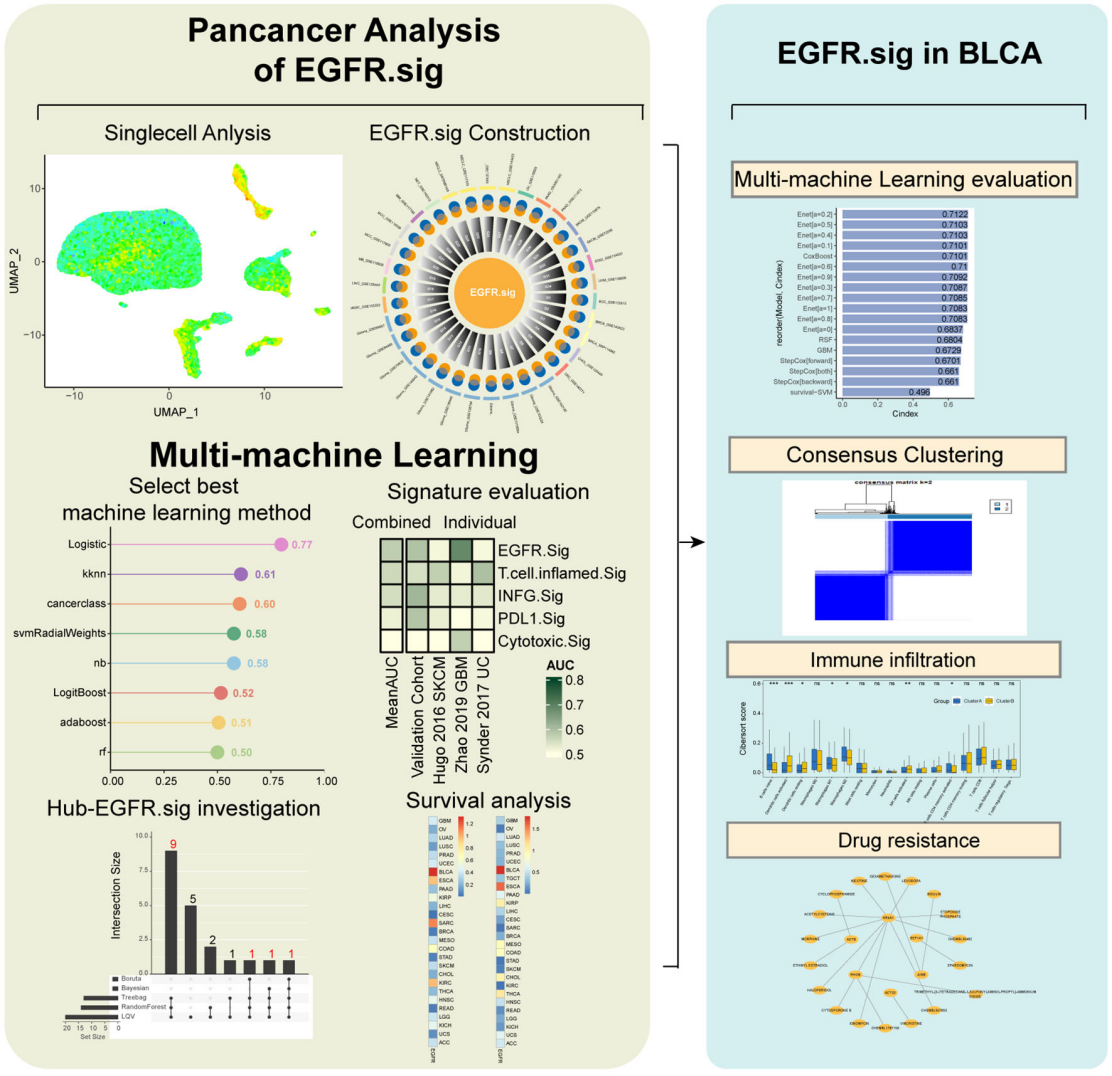
Workflow of this study.

The workflow is presented in **Figure 1**.

### 2.2 ScRNA-Seq data analysis

All scRNA-Seq data analyses and integrations were performed using R software Seurat v4.0.6. Based on the current mainstream practice in the field of scRNA-Seq of tumor tissues, we set strict quality control thresholds, excluding cells with less than 300 genes in a single cell and cells with more than 20% of mitochondrial genes (41). The data normalization and standardization of each sample were achieved by principal component analysis (PCA), and the batch differences between samples were achieved by the “harmony” package. The annotation of major cell types was performed based on well-established canonical marker genes from the TISCH database and relevant literature (23). Detailed procedures can refer to the TISCH portal website (http://tisch.comp-genomics.org/documentation/). Then, we reduced the dimension and visualized the scRNA-Seq data by the uniform manifold approximation and projection (UMAP) algorithm. The R “FindAllMarkers” package was used to identify differentially expressed genes between different cell types. Genes with a log_2_-fold change (log_2_FC) ≥1 and a false discovery rate (FDR) <1e-05 were considered differentially upregulated genes for each cell subtype (23). For malignant tumor cells, Spearman correlation analysis was used to discover the relationship between EGFR-related gene expression and EGFR scores in 34 scRNA-Seq datasets.

### 2.3 Evaluation of clinical efficacy

The main index of clinical outcomes was the objective response rate (ORR). ORR of all cohorts was assessed with the use of Response Evaluation Criteria in Solid Tumors (RECIST) version 1.1, except Hugo 2016, which was assessed with the immune-related RECIST (irRECIST) (42). According to the response status, patients with complete response (CR) and partial response (PR) were grouped as responders (R), while those with stable disease (SD) and progressive disease (PD) were considered nonresponders (NR). Moreover, TN represents treatment-naive patients.

### 2.4 Machine learning and prediction model construction

Eight common ML algorithms were used to train and adjust prediction models of ICI response based on EGFR.Sig. For the support vector machine (SVM), naive bayes (NB), random forest (RF), k-nearest neighbors (KNN), Adaptive Boosting (AdaBoost), logistic regression model (Logistic) and boosted logistic regression (LogiBoost) algorithms, fivefold cross-validation was used for hyperparameter tuning to optimize the performance of the model (43). To ensure robustness, we repeated the optimization process 10 times using different random seeds for each single resampling (44). For cancer class algorithms, which do not require parameters, we directly used the entire training set to train the model. The receiver operating characteristic (ROC) curve and the area under the curve (AUC) were used to evaluate the sensitivity and specificity of the models. The analyses were performed using R packages including caret (v6.0-93), randomForest (v4.7-1.1), e1071 (v1.7-13), nnet (v7.3-18), and adabag (v4.2). Finally, the models constructed with the above algorithms were analyzed by the validation set to screen out the efficacy prediction model in pan-cancer immunotherapy. Furthermore, we compared the predictive efficiency of gene signatures in our study and other previously reported ICI response pan-cancer biomarkers, including INFG.Sig (45), T.cell.inflamed.Sig (45), PDL1.Sig (46) and Cytotoxic.Sig (47)(**Supplementary Table S2**).

To further screen the core EGFR-related immune efficacy genes, we used 5 ML algorithms, namely, Wrapper, learning vector quantization (LQV), RF, bagged decision tree and Bayesian, for the training set to analyze the important role of EGFR-related genes in immunotherapy. The random forest method was configured with 500 decision trees. The Wrapper method employed stepwise feature selection, while the Bayesian approach utilized a naïve Bayes classifier. Both LQV and bagged decision trees adopted the default parameters from the R caret package. For each model, gene importance was ranked based on feature importance scores (e.g., mean decrease in accuracy for random forest, selection frequency for Wrapper, etc.). Finally, the genes that made sense in at least three algorithms were selected as the hub EGFR-related gene signature (Hub-EGFR.Sig).

To explore the relationship between Hub-EGFR.Sig and the prognosis of cancer patients, we constructed Hub-EGFR.Sig-related prediction models with a total of 18 algorithm models using 5 ML algorithms, namely, elastic network (Enet), random survival forest (RSF), generalized boosted regression modelling (GBM), stepwise Cox and survival support vector machine (Survival-SVM). For the Enet model, we tested alpha values ranging from 0 to 1 with increments of 0.1, ultimately selecting the optimal model with alpha=0.2. For the RSF, we configured the following parameters: number of trees (n.tree)=1000, node size (nodesize)=10, splitting rule=logrank test, and proximity calculation using inbag samples. The GBM was implemented with these specifications: number of trees (n.tree)=10000, interaction depth=3, minimum observations in terminal nodes (n.minobsinnode)=10, and shrinkage=0.001. The Stepwise Cox regression was performed with 1000 steps, while the Survival-SVM used gamma.mu=1 as its key parameter. All model algorithms were based on leave-one-out cross-validation, and the prediction performance of the models was evaluated by Harrell’s concordance index (C-index). The optimal algorithm was selected by comparing the C-index values of different ML algorithms.

### 2.5 CRISPR analysis

To explore potential therapeutic targets based on EGFR.Sig, seven published CRISPR screening studies covering melanoma, breast, colon, and renal cancer cell lines were collected, namely, Freeman 2019 (48), Kearney 2018 (49), Manguso 2017 (50), Pan 2018 (51), Patel 2017 (52), Vredevoogd 2019 (53), and Lawson 2020 (54). According to the model cell lines and treatment conditions, these seven CRISPR studies were divided into 17 datasets that evaluated the individual effects of each gene knockout on tumor immunity (40). Data were utilized to identify genes that were more likely to influence the response to immunotherapy across different cancer cell lines.

Generally, the first step of CRISPR screening is to conduct genome-wide CRISPR–Cas9 knockout across various cancer cells. Then, the cells are either cocultured with cytotoxic lymphocytes (CTLs) *in vitro* or implanted in immunodeficient/immunocompetent mice *in vivo*. Finally, ten RNA sequences are used to estimate the sgRNA abundance of the genes studied. By calculating the log-fold changes in sgRNA readings between gene knockout cell lines and control groups, we can understand the effect of gene knockout on cancer under the pressure of CTLs or antitumor immunity (54). This study invokes the normalized z scores from log-fold changes to eliminate the batch effect and compare genes in different CRISPR datasets. The genes were sorted from low to high according to their average z scores across the 17 datasets. The top-ranked genes have lower average z scores, and the lower z scores indicate a better immune response after gene knockout, so these genes were identified as immune resistance genes. Referring to previous studies, the proportion of the first 6% top-ranked immune resistance genes in EGFR.Sig and other published ICI response signatures (CRMA.Sig (55), LRRC15.CAF.Sig (56), IMS.Sig (57), TcellExc.Sig (21), ImmmunCells.Sig (58)) was compared (40).

### 2.6 Survival analysis

We used 1000 iterations of 10-fold cross-validation LASSO-Cox for dimensionality reduction screening. Each patient’s risk score was computed by summing the products of expression levels of selected Hub-EGFR.Sig genes and their corresponding LASSO-Cox regression coefficients, following the formula: risk score = (β_1_ × expression of gene_1_) + (β_2_ × expression of gene_2_) +… + (β_n_ × expression of gene_n_). The cancer patients were divided into a high-risk group and a low-risk group according to the median score to explore the relationship between Hub-EGFR.Sig and prognosis in pan-cancer by Kaplan–Meier (K–M) survival analysis. The “survival” package of R was used to perform survival analyses. Disease-specific survival (DSS) and progression-free interval (PFI) were used in the survival analysis of this study because they can well reflect the effectiveness of clinical treatment (59). We also analyzed the Spearman correlation and drew the correlation heatmap of DSS or PFI through the “cowplot” package and verified it by the “GOSemSim” package.

### 2.7 Molecular subtype identification and characteristic analysis

Consensus clustering using the “ConsensusClusterPlus” package was conducted to identify different populations with EGFR functional phenotypes in BLCA based on Hub-EGFR.Sig. Principal coordinate analysis (PCoA) was used to verify the results of consensus clustering. The immunotherapy response of BLCA patients in different clusters was predicted by Tumor Immune Dysfunction and Exclusion (TIDE, http://tide.dfci.harvard.edu), which is a widely recognized program for evaluating the prognosis of ICI therapy (60, 61). The R “ggpubr” package was used to draw a bar graph of immunotherapy response between different clusters. From Genomic Data Commons (GDC, https://portal.gdc.cancer.gov), we obtained the mutation information of TCGA datasets and analyzed the characteristics with the “maftools” packet. To systematically identify pathway-level alterations associated with the observed mutational profiles, we first identified significantly differentially mutated genes (q-value < 0.1) with high mutation frequencies between two clusters. These selected genes were then subjected to pathway enrichment analysis through hypergeometric testing against two comprehensive pathway databases: the Hallmark gene sets from MSigDB (https://www.gsea-msigdb.org/gsea/msigdb) and the Reactome database (https://reactome.org/). All analyses were performed using the clusterProfiler R “clusterProfiler” package, with statistical significance of pathway enrichment determined after Benjamini-Hochberg multiple testing correction (FDR < 0.05). Moreover, the Drug Gene Interaction database (DGIdb) was used to predict the mutant gene druggability of different subtypes.

### 2.8 Immune cell infiltration analysis

The infiltration of immune cells in the TIME was evaluated by CIBERSORT (https://cibersort.stanford.edu/) and single-sample gene set enrichment analysis (ssGSEA). For CIBERSORT, we first uploaded gene expression data to the CIBERSORT portal and then evaluated the infiltration of immune cells in samples based on the gene expression features of 22 known immune cell subtypes (62). For ssGSEA, the enrichment scores calculated by ssGSEA were used to express the relative abundance of immune cell infiltration in each sample. Finally, we used the “ggplot2” and “pheatmap” packages to present the results.

### 2.9 Network regulation and interaction analysis

We searched the regulatory relationship and network interaction of Hub-EGFR.Sig in the miRTarBase v8.0 (https://mirtarbase.cuhk.edu.cn/) and ENCODE (https://www.encodeproject.org/) databases, and the mRNA–miRNA and mRNA-TF network information was collected. Then, we predicted the interaction between proteins corresponding to the key genes through the online database STRING (https://string-db.org). All interactive networks were visualized with Cytoscape software.

### 2.10 Cells and cell culture

Human ureteral epithelial immortalized cell line SV-HUC-1 and human bladder cancer cell line 5637 were purchased from Wuhan Servicebio Technology Co., Ltd (Wuhan, China). Cells were cultured in a 37°C, 5% CO_2_ incubator using special media purchased from Servicebio, Inc. (GZ12301, GZ11703).

### 2.11 Western blotting

Total protein was obtained with RIPA lysis buffer (P0013B, Beyotime, Shanghai, China). BCA Protein Assay Kit (P0012, Beyotime, Shanghai, China) was employed to measure protein concentration. Proteins were subjected to SDS-page for separation and then transferred to PVDF membranes (0.22 µm, Millipore). 5% milk blocking buffer (P30500, NCM Biotech, Soochow, China) was used to block nonspecific binding sites. The membranes were incubated with specific primary antibodies overnight at 4°C. Subsequently, washed membranes were incubated with corresponding secondary antibodies for 1 h at room temperature. ECL Chemiluminescence kit was purchased from Mishu Biotechnology (MI00607B, Xi ‘an, China). The primary antibodies included ABCA7(YP-mAb-05349, UpingBio, China), HOOK2 (YP-mAb-10034, UpingBio, China), VMP1 (F1488, Selleck, China), JUNB (F0578, Selleck, China), ACTG1(YP-mAb-06774, UpingBio, China), RHOB (YP-mAb-16239, UpingBio, China). The second antibodies included goat anti-mouse IgG-HRP (abs20039, Absin, China) and goat anti-rabbit IgG-HRP (abs20040, Absin, China).

### 2.12 The Human Protein Atlas analysis

The Human Protein Atlas (HPA, https://www.proteinatlas.org/) is a human proteome atlas database containing information on the protein distribution of human tissues and cells. To analyze the differential expression of ABCA7, HOOK2, VMP1, JUNB, ACTG1 and RHOB at the protein level, we downloaded immunohistochemical images of bladder tumor tissues with their corresponding normal tissues from HPA.

### 2.13 Statistical analysis

All data processing and analysis were performed with R software (version 4.0.2). For comparisons of continuous variables between the two groups, the statistical significance of normally distributed variables was estimated with an independent Student’s t test, and differences between variables that were not normally distributed were analysed with the Mann–Whitney U test (i.e., the Wilcoxon rank-sum test). The chi-square test or Fisher’s exact test was used to compare and analyze the statistical significance between the two groups of categorical variables. *P*<0.05 was used as the criterion for significantly different results if there were no special notes.

## 3 Results

### 3.1 Relationship between EGFR-related genes and tumor immunotherapy efficacy

**Figure 2:**
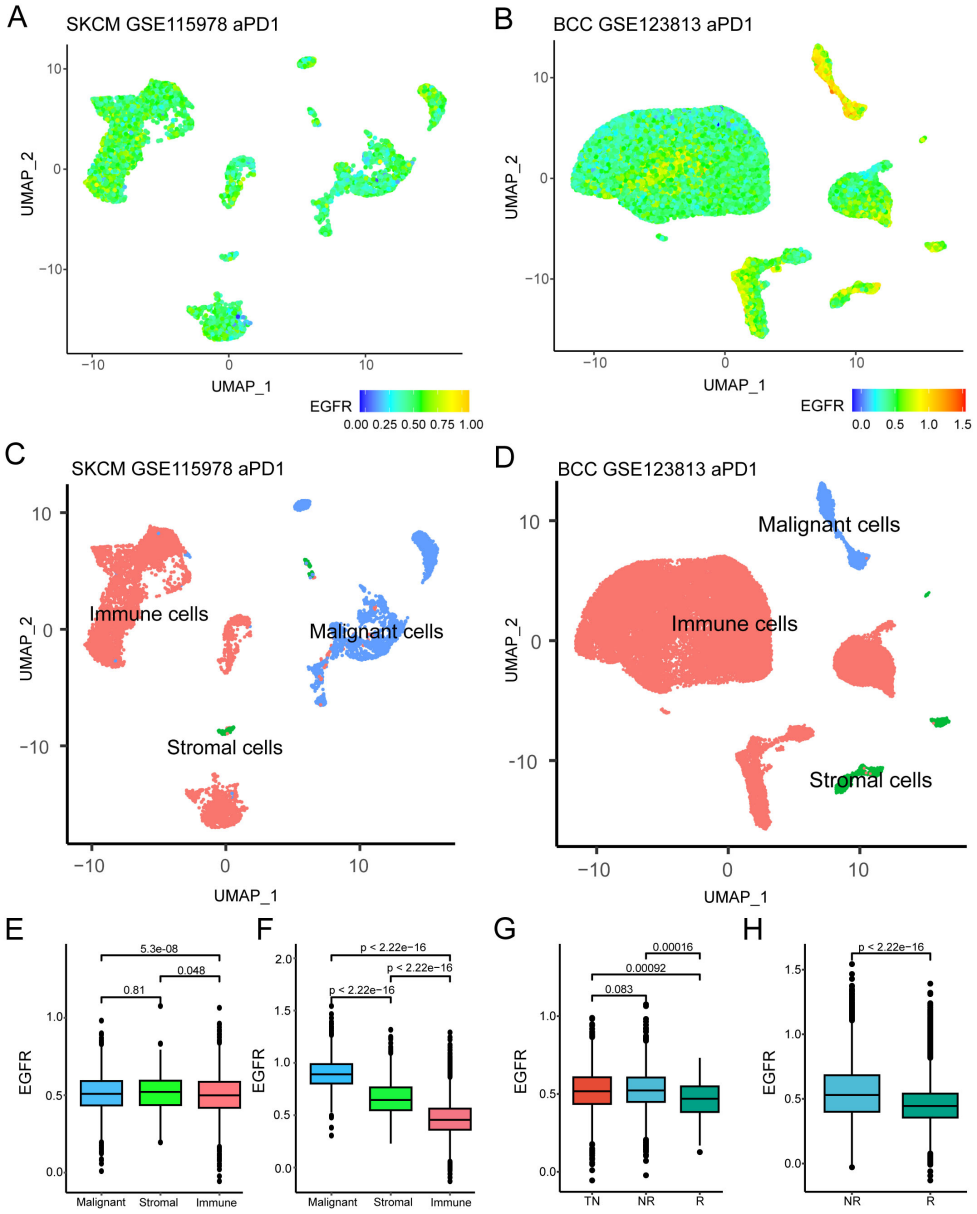
Relationship between EGFR related genes and immunotherapy efficacy. **(A, B)** UMAP plot of EGFR related genes scores in GSE115978 and GSE123813. **(C, D)** UMAP plot of different cell subtypes in GSE115978 and GSE123813. **(E, F)** Differences in EGFR related gene scores among different cell subtypes in GSE115978 and GSE123813 (Wilcoxon test). **(G, H)** EGFR related genes scores of patients with different immunotherapy responses in GSE115978 and GSE123813(Wilcoxon test). Abbreviation: SKCM, skin cutaneous melanoma; BCC, basal cell carcinoma; NR, non-responders; R, responders; TN, treatment naïve patients.

Two scRNA-Seq GEO datasets with clear efficacy of tumor immunotherapy were downloaded to evaluate the relationship between EGFR-related genes and tumor immunotherapy outcomes. After quality control, 33 samples were included in the GSE115978 cohort (melanoma, TN=16, NR=16, R=1), and 10 patients were included in the GSE123813 cohort (BCC, NR=4, R=6). First, we comprehensively presented the distribution of EGFR scores (defined as the ssGSEA-derived enrichment score of the EGFR gene set) among single-cell samples and found that the distribution was uneven (**Figures 2A, B**). Considering the presence of different cell types in tumor tissues, we marked these cells and found that they could be roughly divided into three categories: immune cells, stromal cells and malignant cells (**Figures 2C, D**). Then, we investigated the differences in EGFR scores among different cell subtypes. The results demonstrate a statistically significant difference in EGFR scores between malignant and immune cells (**Figures 2E, F**). This discrepancy was particularly pronounced in the GSE123813 dataset, where malignant cells demonstrated significantly elevated EGFR scores while immune cells showed markedly lower scores (mean=0.89 vs 0.47, **Figure 2F**). Subsequent analysis revealed that the EGFR scores of patients in GSE115978 who were untreated or did not respond to immunotherapy were higher than those of patients who responded to immunotherapy (*P* < 0.001, **Figure 2G**). Similar results were also observed in GSE123813 (*P* < 0.001, **Figure 2H**). These results indicated that the EGFR-related genes may have a potential connection with the response to immunotherapy in cancer patients.

### 3.2 Development of EGFR.Sig based on pan-cancer scRNA-Seq analysis

**Figure 3:**
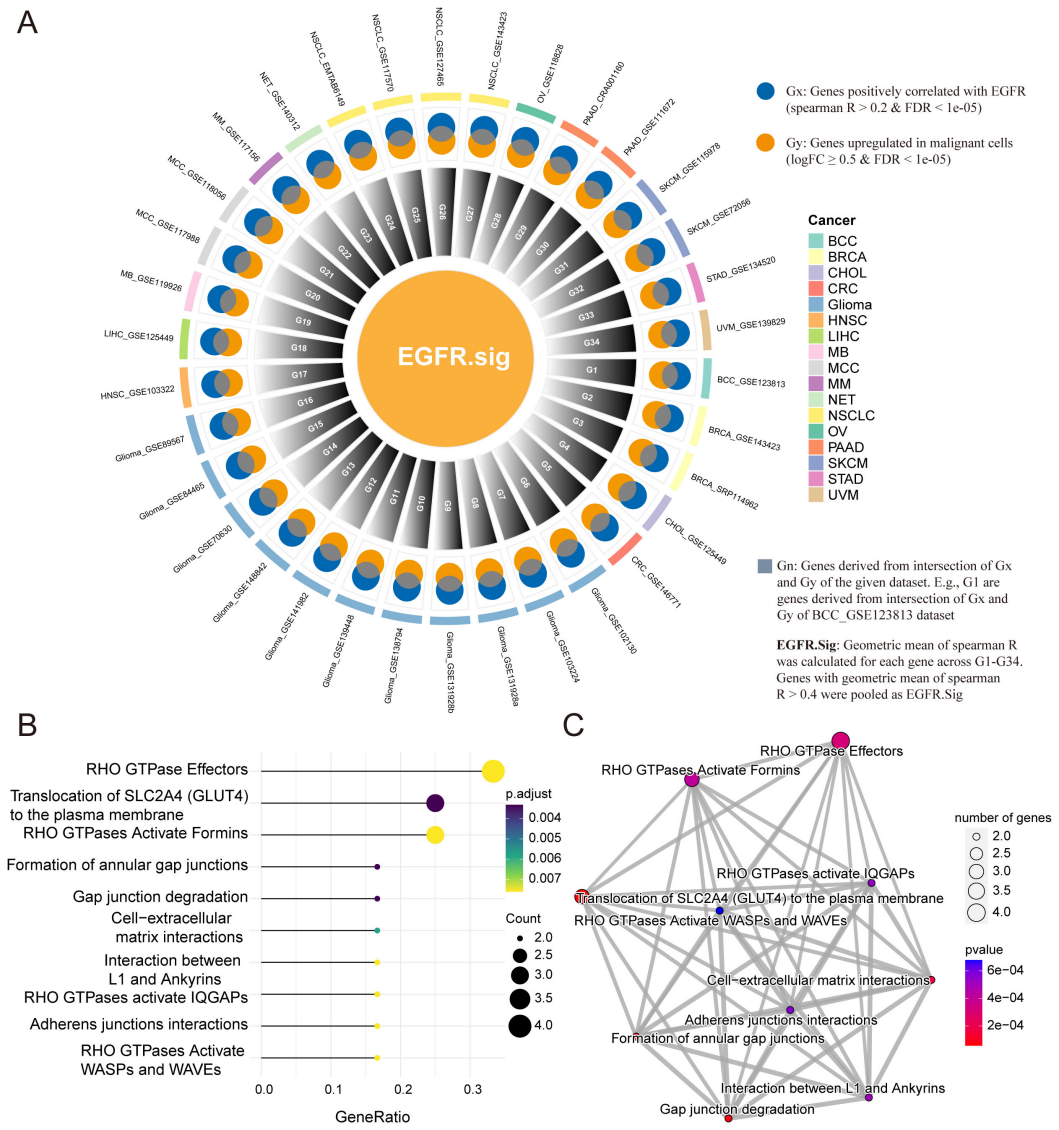
Development of EGFR related gene signature and enrichment analysis. **(A)** Circle diagram depicting the development of EGFR.Sig. **(B)** Top 10 enriched Reactome pathways of genes in EGFR.Sig. **(C)** The network of enriched Reactome pathways of EGFR.Sig. The colored dots indicate the corresponding pathway, the size of the dots indicates the number of enriched genes in the pathway, and the depth of the color indicates the *P* value.

As there were differences in the EGFR scores of cancer patients with different immunotherapy responses, we hypothesized that the evaluation of EGFR.Sig expression levels could predict the efficacy of immunotherapy to some extent. Therefore, 34 pan-cancer scRNA-Seq datasets were included to screen important EGFR-related genes for EGFR.Sig (**Figure 3A**). First, Spearman correlation was performed to analyze the relationship between EGFR-related genes and EGFR scores in malignant tumor cells across 34 scRNA-Seq datasets. If there was a positive correlation (Spearman R > 0.2 and FDR < 0.05), the gene was labelled Gx. Then, genes highly expressed in malignant tumor cells were screened and marked as Gy (logFC ≥ 0.5 and FDR < 0.05). Gn was generated by the intersection of Gx and Gy in each dataset to identify the upregulated genes in specific tumors that were positively related to EGFR. For example, Gx and Gy were screened out in the first scRNA-Seq dataset, and their common genes make up G1. Finally, the geometric mean of Spearman R was calculated, and genes with a geometric mean R > 0.4 (moderate to strong correlation) in G1-G34 were merged as EGFR.Sig (**Supplementary Table S3**). Furthermore, we investigated the biological functions of EGFR.Sig. The EGFR.Sig enrichment pathways mainly included RHO GTPase effectors, translocation of SLC2A4 and RHO GTPases activating formins (**Figure 3B**), and there were interrelations and interaction networks among these functional pathways (**Figure 3C**).

### 3.3 Potential link between EGFR.Sig and immune suppression across cancers

**Figure 4:**
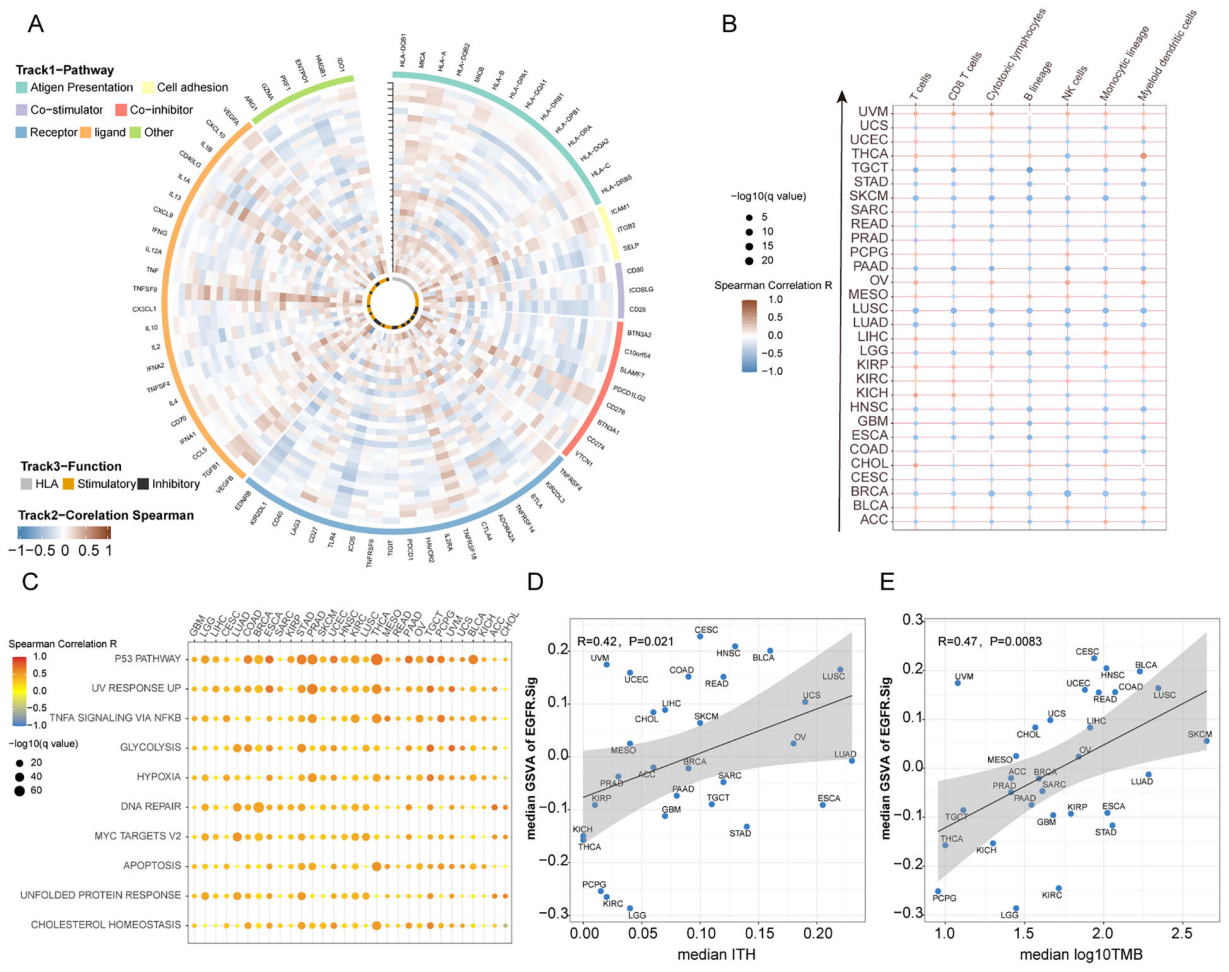
Potential links between EGFR.Sig and immune resistance in pan-cancer. **(A)** Circos plot depicting the correlation between the expression levels of EGFR.Sig and immune related genes in pan-cancer. Types of cancer from inner to outer rings refer to the y-axis of plot **(B)** B. Heatmap depicting the correlation between EGFR.Sig and the infiltration of immune cells across multiple cancer types. **(C)** Correlation between EGFR.Sig and hallmark pathways. **(D)** Correlation between median EGFR.Sig score and median ITH score across cancer types. **(E)** Correlation between median EGFR score and median TMB score across cancer types.

To further explore the relationship between EGFR.Sig and the effect of cancer immunotherapy, we comprehensively analysed the correlation between EGFR.Sig and 75 immune-related genes previously published in the pan-cancer TCGA cohort (**Supplementary Table S4**) (28). A generally correlation between EGFR.Sig and most of the immune-related genes across 30 different cancers was observed (**Figure 4A**). Then, we evaluated the CIBERSORT infiltration abundance of immune cells in the TIME across TCGA pan-cancer cohort. The results demonstrated that EGFR.Sig was negatively correlated with the infiltration of various immune-promoting cells, including CD8^+^ T cells, NK cells and macrophages, in different cancer types (**Figure 4B**). Next, we analysed the hallmark pathways associated with EGFR.Sig in the TCGA pan-cancer dataset by calculating Spearman correlation coefficients between EGFR.Sig expression and the enrichment scores (obtained by ssGSEA calculations) of each hallmark pathway. In our analysis, we observed that EGFR.Sig showed a positive correlation with certain immune-related biological pathways (e.g., P53, UV response up and DNA repair pathway) (**Figure 4C**) (63–65). Furthermore, the relationship between EGFR.Sig and ITH, which is a stem-related trait associated with tumor immunosuppression (66), was analysed. Similarly, ITH was positively correlated with EGFR.Sig in pan-cancer (R = 0.42, *P* = 0.021, **Figure 4D**). Moreover, the TMB, a well-known molecular marker related to immunotherapy efficacy, was also detected to have a similar positive association with EGFR.Sig (R = 0.47, *P* = 0.0083, **Figure 4E**). In summary, EGFR.Sig was observed to be negatively correlated with the tumor immune response.

### 3.4 Immune response prediction by EGFR.Sig

**Figure 5:**
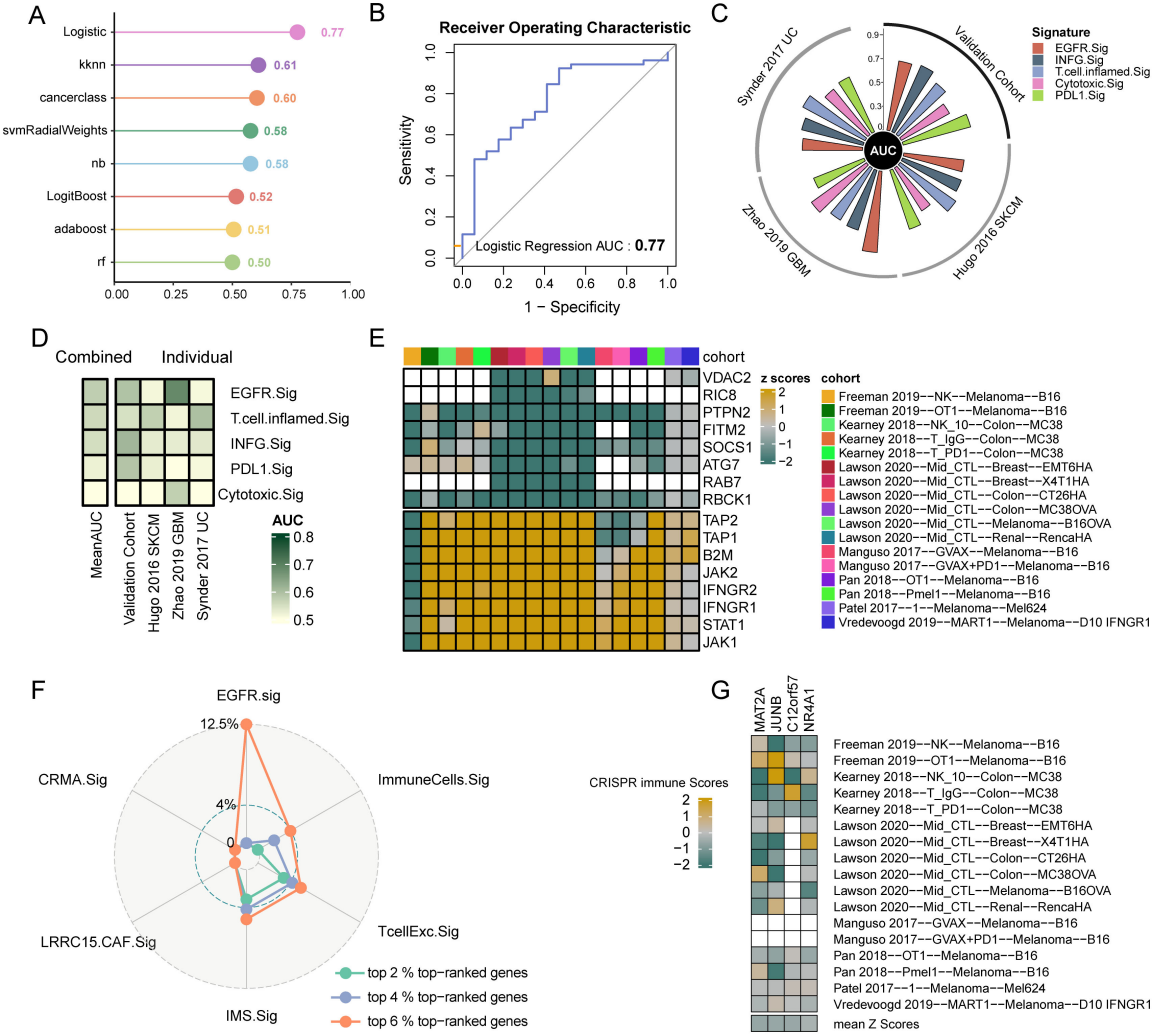
Predictive value to immunotherapy and potential therapeutic targets of EGFR.Sig. **(A)** AUC of the prediction models constructed by eight ML algorithms in the validation set. **(B)** ROC plot of Logistic regression algorithm. **(C)** The performance of immune response signatures in independent external validation set. The vertical axis indicated AUC values. The independent external validation set comprises three different cohorts including Hugo 2016 SKCM, Zhao 2019 GBM and Synder 2017 UC. **(D)** The predictive value of immune response signatures. The signatures are ordered by mean AUC from top to bottom. **(E)** Genes ranking at top and bottom according to their average z-scores across 17 CRISPR datasets. Top-ranked (bottom-ranked) genes associated with immune resistance (sensitive) and the anti-tumor immune response is better (worse) after knockout. Blank squares indicate the missing values of gene data in the corresponding cohorts. **(F)** Comparing the percentage of top-ranked genes among immune response signatures. **(G)** The z-scores of the four screened EGFR.Sig genes in the different CRISPR datasets. CRISPR immune scores represent the average normalized z-score of each gene across 17 CRISPR datasets. AUC, area under the curve; Sig, signature.

To effectively predict the immunotherapy response of cancer patients through EGFR.Sig, bulk RNA-Seq cohorts with clear immunotherapy response information were identified and assessed. The cohorts were divided into a training set (n = 618) and a validation set (n = 154). First, we used eight ML algorithms to construct and optimize prediction models for immunotherapy efficacy and verified the models by ROC. By comparing the AUCs of different algorithm models, we found that the logistic regression model had better predictive efficiency than the other algorithms, with an AUC of 0.77 (**Figures 5A, B**). Then, we compared the predictive performance of EGFR.Sig with that of previously published gene signatures in another independent validation set to validate the superiority of EGFR.Sig. The results demonstrated that that EGFR.Sig exhibited superior predictive performance compared to other published gene signatures in the Zhao 2019 GBM cohort, while maintaining consistently robust predictive power relative to existing signatures across multiple independent validation cohorts (**Figure 5C**, **Supplementary Table S2**). As revealed in the heatmap, the average AUC of EGFR.Sig ranked at the top of the published gene signatures (**Figure 5D**), which indicated a better ability of EGFR.Sig to predict the immunotherapy response, especially for the Zhao 2019 GBM cohort. Taken together, the EGFR.Sig predictive models developed with the logistic regression algorithm can effectively predict the response of cancer patients to immunotherapy.

### 3.5 Screening potential therapeutic targets associated with EGFR.Sig

Seven CRISPR cohorts including 17 CRISPR datasets were systematically collected to further explore potential therapeutic targets, and 22505 knockout genes with immune response data were ranked according to the average z scores. Lower z-scores indicate that gene knockout enhances tumor cell susceptibility to immune-mediated killing. Therefore, the top genes were considered immune resistance genes, while those at the bottom were considered immune sensitive genes (**Figure 5E**). Then, we calculated the proportion of the first 6% top-ranked genes in EGFR.Sig and other published ICI response signatures. The results showed that there were four top 6% CRISPR genes, which accounted for 12.5% of EGFR.Sig, and the proportion of immune resistance genes (6% top-ranked genes) in EGFR.Sig was much higher than that of other gene signatures (**Figure 5F**). The four identified immune resistance genes were MAT2A, JUNB, C12orf57 and NR4A1, which were verified in the CRISPR dataset and were thought to promote antitumor immunotherapy after knockout (**Figure 5G**). Therefore, these genes may be meaningful targets in synergy with immunotherapy for cancer patients.

### 3.6 Identification of prognostic genes in EGFR.Sig

**Figure 6:**
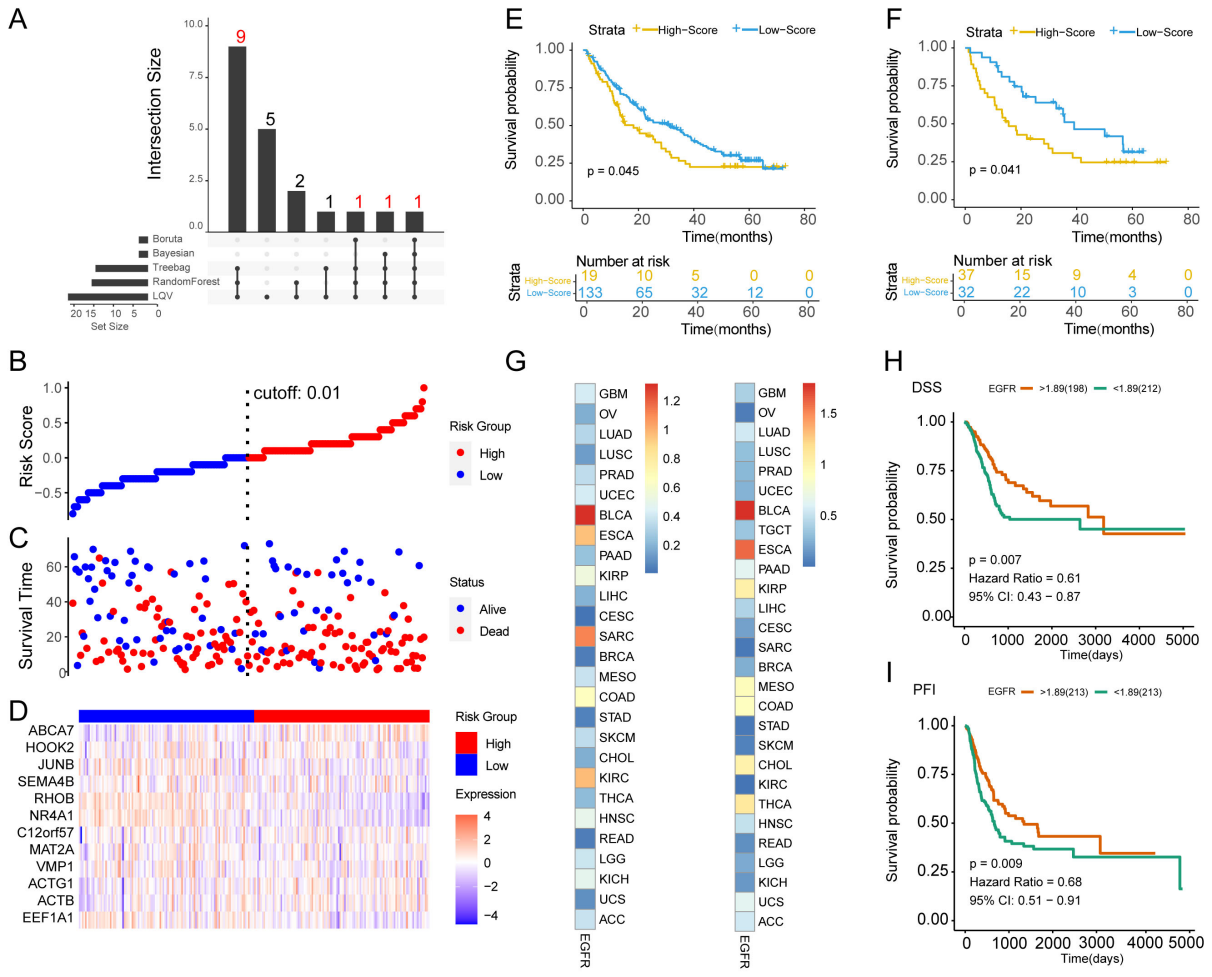
Identification of the Hub-EGFR.Sig and survival analysis in pan-cancer. **(A)** Intersection of the core genes in 5 different ML algorithms. **(B)** Distribution of patients according to the Hub-EGFR.Sig risk score from low to high. Patients were divided into high and low risk groups with 0.01 as the best cutoff value. **(C)** Survival time and status distribution of patients in high and low risk groups. **(D)** Heatmap of Hub-EGFR.Sig expression in high and low risk groups. **(E, F)** K-M survival analysis of the high and low risk groups in the training set and validation set (Log-rank test). **(G)** The relationship between Hub-EGFR.Sig and DSS or PFI in different cancers. The p values in the figure are converted, and the darker the colour means the lower the *P* value. **(H, I)** DSS and PFI of high and low Hub-EGFR.Sig score groups in bladder cancer (Log-rank test). DSS, disease specific survival; PFI, progression free interval.

To further screen the core genes in EGFR.Sig and explore its relationship with the prognosis of cancer patients, five ML algorithms were used to analyze the importance of genes for tumor immunotherapy response in the training set (**Supplementary Figures S1A-E**). The genes determined to be most closely related to tumor immunotherapy efficacy in at least three algorithms were ABCA7, ACTB, ACTG1, C12orf57, EEF1A1, HOOK2, JUNB, MAT2A, NR4A1, RHOB, SEMA4B and VMP1 (**Supplementary Figure S1F**), and they were considered the hub genes comprising Hub-EGFR.Sig (**Figure 6A**). Furthermore, cancer patients in the total ICI cohorts were divided into two subgroups according to the risk scores of Hub-EGFR.Sig (**Figures 6B, C**), and Hub-EGFR.Sig gene expression varied between the two groups (**Figure 6D**). To understand the relationship between Hub-EGFR.Sig and the immunotherapy prognosis of pan-cancer, PFI were calculated, and the results indicated that patients with higher scores of Hub-EGFR.Sig had a worse prognosis in the training set (*P* = 0.045, **Figure 6E**). As expected, similar results were also observed in the verification set (*P* = 0.041, **Figure 6F**).

### 3.7 Protein interaction networks of Hub-EGFR.Sig

In the above combined ICI treatment cohort, we investigated the expression of immunogenic death (ICD)-related genes that are closely related to the efficacy of immunotherapy (67). The expression of 13 ICD-related genes showed significant differences between the R and NR cancer patients treated with ICIs. Among them, BAX, CALR, FOXP3, HSP90AA1, IFNB1, IL6, PIK3CA and TLR4 were highly expressed in the NR group, while CXCR3, EIF2AK3, ENTPD1, IFNG and PRF1 were highly expressed in the R group (**Supplementary Figures S2A-M**). To further analyze the interaction between Hub-EGFR.Sig and ICD genes, we constructed PPI networks using the STRING database. Finally, we obtained a regulatory network of 38 nodes with a wide range of connections (**Supplementary Figure S2N**), which reflected the universal and close relationship of Hub-EGFR.Sig and ICD in the immune response of pan-cancer. In addition, we constructed the mRNA–miRNA network and mRNA-TF network to reveal potentially regulated molecules of Hub-EGFR.Sig. There were 49 interactions, including 11 genes in Hub-EGFR.Sig and 17 miRNAs from the miRTarBase v8.0 database (**Supplementary Figure S2O**). Among them, EEF1A1 interacted with 11 miRNAs, MAT2A and ACTB interacted with 6 miRNAs, and RHOB interacted with 5 miRNAs, showing the most common connection. Similarly, the constructed mRNA-TF network included 98 interactions, which consisted of all the Hub-EGFR.Sig genes and 14 TFs from the ENCODE database (**Supplementary Figure S2P**). The Hub-EGFR.Sig genes that interacted most widely with TFs were ABCA7, HOOK2, and JUNB, which interacted with all 14 TFs, and NR4A1, which interacted with 10 TFs. In summary, these findings provide an important reference for future studies on the regulatory mechanism of Hub-EGFR.Sig in cancer immunotherapy.

### 3.8 Landscape of Hub-EGFR.Sig in pan-cancer

We systematically reviewed the Hub-EGFR.Sig scores in TCGA pan-cancer datasets. As expected, Hub-EGFR.Sig presented stable enrichment scores across multiple cancer types, of which LUAD, LUSC and BLCA showed higher scores (**Supplementary Figure S3A**). In addition, various immune cells in the TIME, such as CD4 memory T cells, M2 macrophages and resting mast cells, were negatively correlated with Hub-EGFR.Sig in most types of cancer, while follicular helper T cells, active mast cells, eosinophils and plasma cells were positively correlated (**Supplementary Figure S3B**). Then, the correlation between Hub-EGFR.Sig and microsatellite instability (MSI) was analysed in pan-cancer samples. The results suggested that there was a positive correlation between Hub-EGFR.Sig and MSI in most cancer types but a negative correlation in adrenocortical carcinoma and pancreatic cancer (**Supplementary Figure S3C**). Furthermore, we analysed the correlation between the 12 genes in Hub-EGFR.Sig and MSI (**Supplementary Figure S3D**). The results showed that C17orf57 and SEMA4B were positively correlated with MSI, while RHOB presented a negative correlation in colon cancer. In BLCA, HOOK2 and ABCA7 were positively correlated with MSI, while SEMA4B and EEF1A1 were negatively correlated. Moreover, SEMA4B and HOOK2 were significantly associated with MSI in more than 10 cancers. In summary, Hub-EGFR.Sig plays an important role in the TIME of pan-cancer, but there is heterogeneity in different types of cancer.

### 3.9 Relationship between Hub-EGFR.Sig and prognosis in different tumor types

Considering the heterogeneous function of Hub-EGFR.Sig in pan-cancer, we subsequently performed survival analysis across tumor types. Correlation analysis demonstrated that the relationship between Hub-EGFR.Sig and DSS or PFI varied in different cancers (**Figure 6G**). Notably, Hub-EGFR.Sig showed the closest correlation with BLCA both in DSS (HR = 0.61, *P* = 0.007, **Figure 6H**) and PFI (HR = 0.68, *P* = 0.009, **Figure 6I**). Next, we comprehensively evaluated the 12 genes in Hub-EGFR.Sig and found that the expression levels of 6 genes had a significant effect on both the DSS and PFI of BLCA patients (**Supplementary Figures S4A-X**). In the results of K–M analysis, higher expression of ABCA7 (**Supplementary Figures S4A, B**), HOOK2 (**Supplementary Figures S4K, L**), JUNB (**Supplementary Figures S4M, N**), RHOB (**Supplementary Figures S4S, T**), and VMP1 (**Supplementary Figures S4W, X**) indicated better survival viability, while higher expression of ACTG1 was associated with poorer survival (**Supplementary Figures S4C, D**). Altogether, there is a significant association between Hub-EGFR.Sig and the prognosis of BLCA.

### 3.10 Prognostic model construction, subtype distinction and immune cell infiltration of BLCA based on Hub-EGFR.Sig

**Figure 7:**
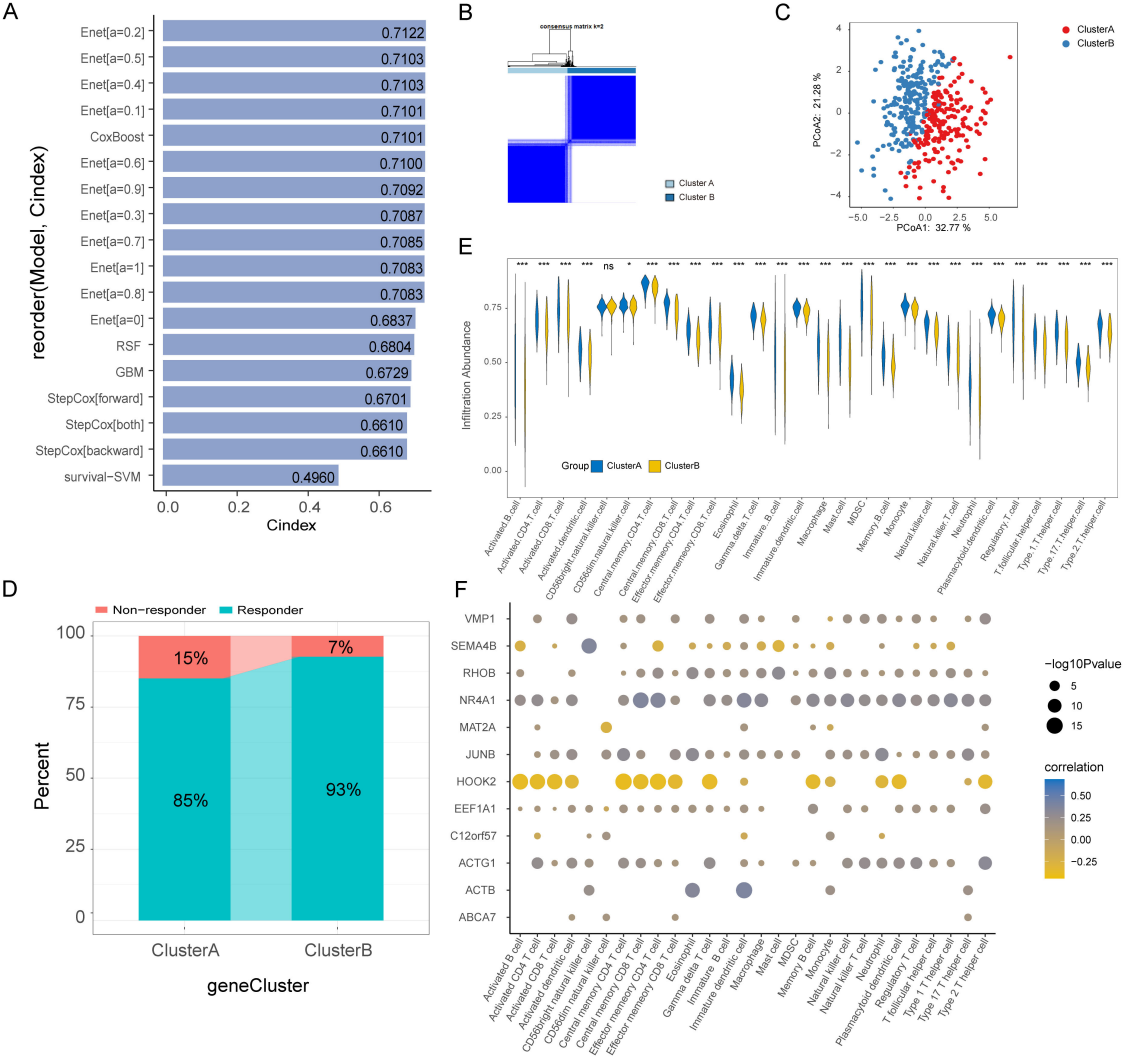
Prognostic model, molecular subtypes and immune cells infiltration (ssGSEA) of bladder cancer based on Hub-EGFR.Sig. **(A)** C-index of the 18 ML algorithm models based on Hub-EGFR.Sig to predict the prognosis of patients in BLCA. **(B)** Consensus cluster analysis of patients in BLCA. **(C)** PCoA analysis of patients in BLCA. **(D)** Immunotherapy response predicted by TIDE in two clusters of BLCA. **(E)** Immune cell infiltration in the two Hub-EGFR.Sig subtypes patients of BLCA. Student’s t-test, *P<0.05, **P<0.01, ***P<0.001 and ns means no significant. **(F)** Correlation analysis between genes in Hub-EGFR.Sig and immune cell abundance in BLCA.

In the TCGA-BLCA dataset, we applied 5 ML algorithms and a total of 18 algorithm models to construct a prognostic model based on Hub-EGFR.Sig. The C-index indicated that Hub-EGFR.Sig in most of the prognostic models had a satisfactory performance in predicting the prognosis of BLCA, among which the Enet[a=0.2] algorithm had the best prediction performance (C-index = 0.7122, **Figure 7A**). Then, we conducted consensus clustering to identify the Hub-EGFR.Sig-related BLCA subtypes. With a consensus matrix of k = 2, patients could be divided into two distinct subgroups named Cluster A and Cluster B (**Figure 7B**). The stability of consensus clustering was validated by PCoA (**Figure 7C**). Further exploration revealed that patients in Cluster B were more likely to benefit from immunotherapy predicted by TIDE (**Figure 7D**). Next, ssGSEA was used to evaluate the differences in immune cell infiltration between the two clusters (**Supplementary Table S5**). Except for CD56-bright natural killer cells, which had no significance, the results demonstrated that the levels of all the other immune cells in Cluster A patients were higher than those in Cluster B patients (**Figure 7E**). We further calculated the correlation between Hub-EGFR.Sig and the content of immune cells separately, and the results showed that most of the Hub-EGFR.Sig genes were significantly correlated with the infiltration of immune cells (**Figure 7F**). Among them, HOOK2 was negatively correlated with the abundance of most immune cells, while NR4A1, JUNB and ACTG1 were positively associated with the abundance of immune cells, and their correlation intensities were all above 0.25. CIBERSORT was also utilized to evaluate the infiltration status of 22 immune cells (**Supplementary Figure S5A**). The results showed that the infiltration abundance of naive B cells, M1 macrophages, M2 macrophages and activated memory CD4^+^ T cells in Cluster A patients was significantly higher than that in Cluster B patients, while the infiltration abundance of dendritic cells and NK cells in Cluster B patients was significantly higher than that in Cluster A patients (**Supplementary Figure S5B**). Heatmap presented the immune cell infiltration abundance of different clusters in BLCA (**Supplementary Figure S5C**). Correlation analysis demonstrated that some genes in Hub-EGFR.Sig were related to the infiltration of immune cells in BLCA. For example, SEMA4B was negatively correlated with the content of naive B cells (r = -0.3, *P* < 0.001, **Supplementary Figure S5D**), ACTB was positively related to M1 macrophages (r = 0.29, *P* < 0.001, **Supplementary Figure S5E**) and activated memory CD4^+^ T cells (r = 0.38, *P* < 0.001, **Supplementary Figure S5F**), while HOOK2 was negatively correlated with activated memory CD4^+^ T cells (r = -0.26, *P* < 0.001, **Supplementary Figure S5G**).

### 3.11 Hub-EGFR.Sig-associated mutation characteristics and drug sensitivity analysis

**Figure 8:**
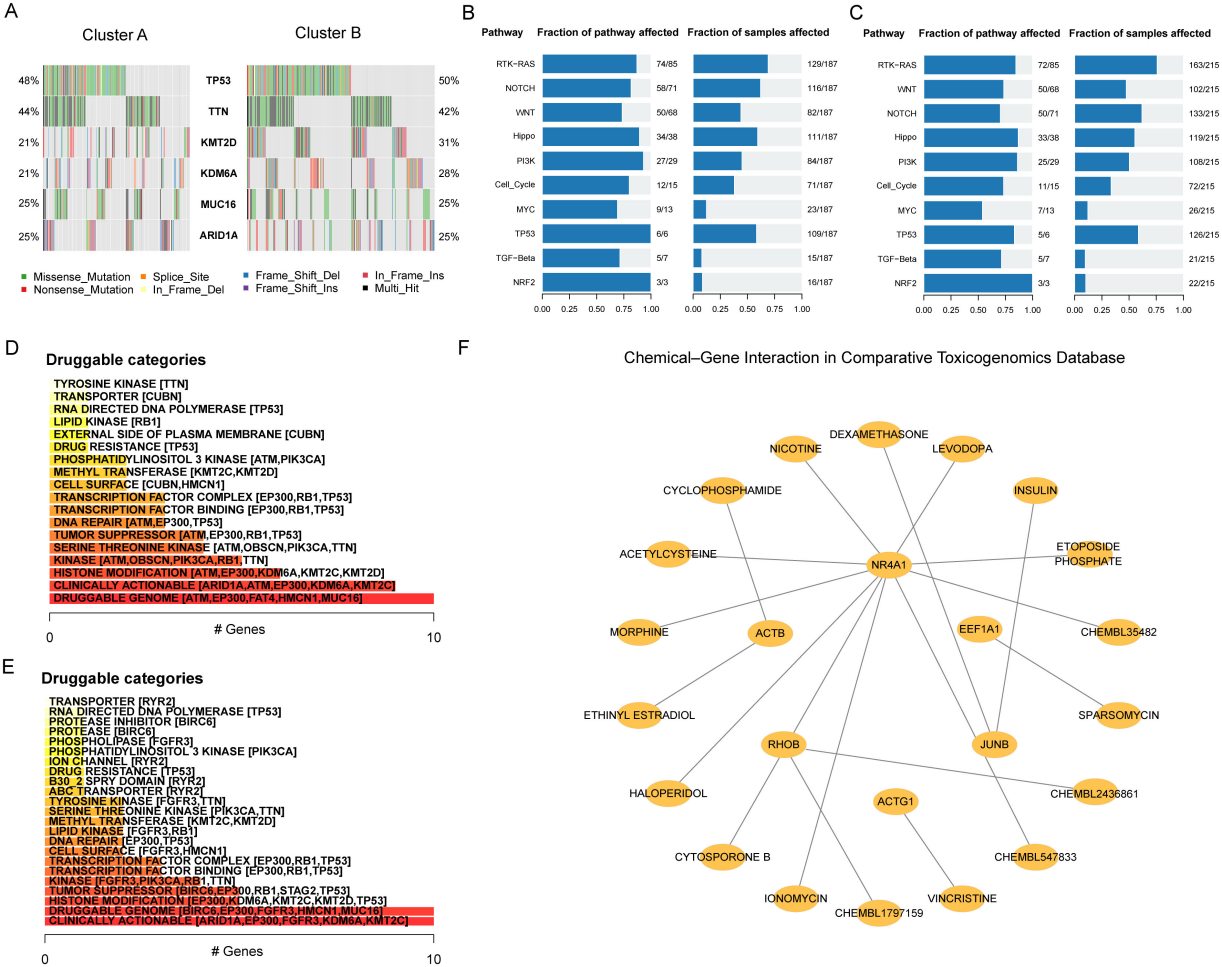
Hub-EGFR.Sig associated mutation characteristics and drug sensitivity analysis. **(A)** Mutant genes landscape in the two Hub-EGFR.Sig subtypes of BLCA. **(B, C)** Biological functions pathways affected by mutations in Cluster A and Cluster B. **(D, E)** Categories of potentially druggable genes in Cluster A and Cluster B. The horizontal axis is the gene counting in the category. Following each category are the top 5 genes in parentheses, and all are shown if less than 5 genes in the category. **(F)** Drug-gene interaction network of Hub-EGFR.Sig and potentially sensitive drugs in BLCA. Nodes without interaction have been removed.

To screen drugs to potentially combine with immunotherapy strategies for BLCA, we further investigated the mutation characteristics in patients with BLCA. TP53, TTN, KMT2D, KDM6A, MUC16, and ARID1A were found to have high mutation frequencies in both clusters (**Figure 8A**). Among them, the mutation frequencies of TP53 were the highest, which were 48% in Cluster A and 50% in Cluster B. Moreover, the mutation frequencies of KMT2D and KDM6A were higher in Cluster B than in Cluster A. For the biological function changes caused by mutations, the top pathways were the RTK-RAS, NOTCH and WNT signaling pathways in Cluster A (**Figure 8B**) and RTK-RAS, WNT and NOTCH signaling pathways in Cluster B (**Figure 8C**). Next, we analysed the druggability drug-gene interaction of the mutant genes in the two clusters through the DGIdb database. The results indicated that the most likely druggable category in Cluster A was the Druggable Genome, in which the first five potential druggable genes were ATM, EP300, FAT4, HMCN1 and MUC16 (**Figure 8D**). The most druggable category in Cluster B was Clinically Actionable, which contained ARID1A, EP300, FGFR3, KDM6A and MT2C as the first five potential druggable genes (**Figure 8E**). Finally, the Comparative Toxicogenomics Database (CTD) was used to identify potentially effective drugs or molecular compounds for Hub-EGFR.Sig in BLCA. The drug-gene interaction network demonstrated that 5 Hub-EGFR.Sig genes were in the interactive network, and 18 drugs or molecular compounds, including etoposide phosphate, sparsomycin and vincristine, had effects on them in BLCA to varying degrees (**Figure 8F**). Although more clinical trials are needed, these findings provide important clues for the future development of therapeutic drugs for BLCA.

### 3.12 Validation of key protein expression levels

**Figure 9:**
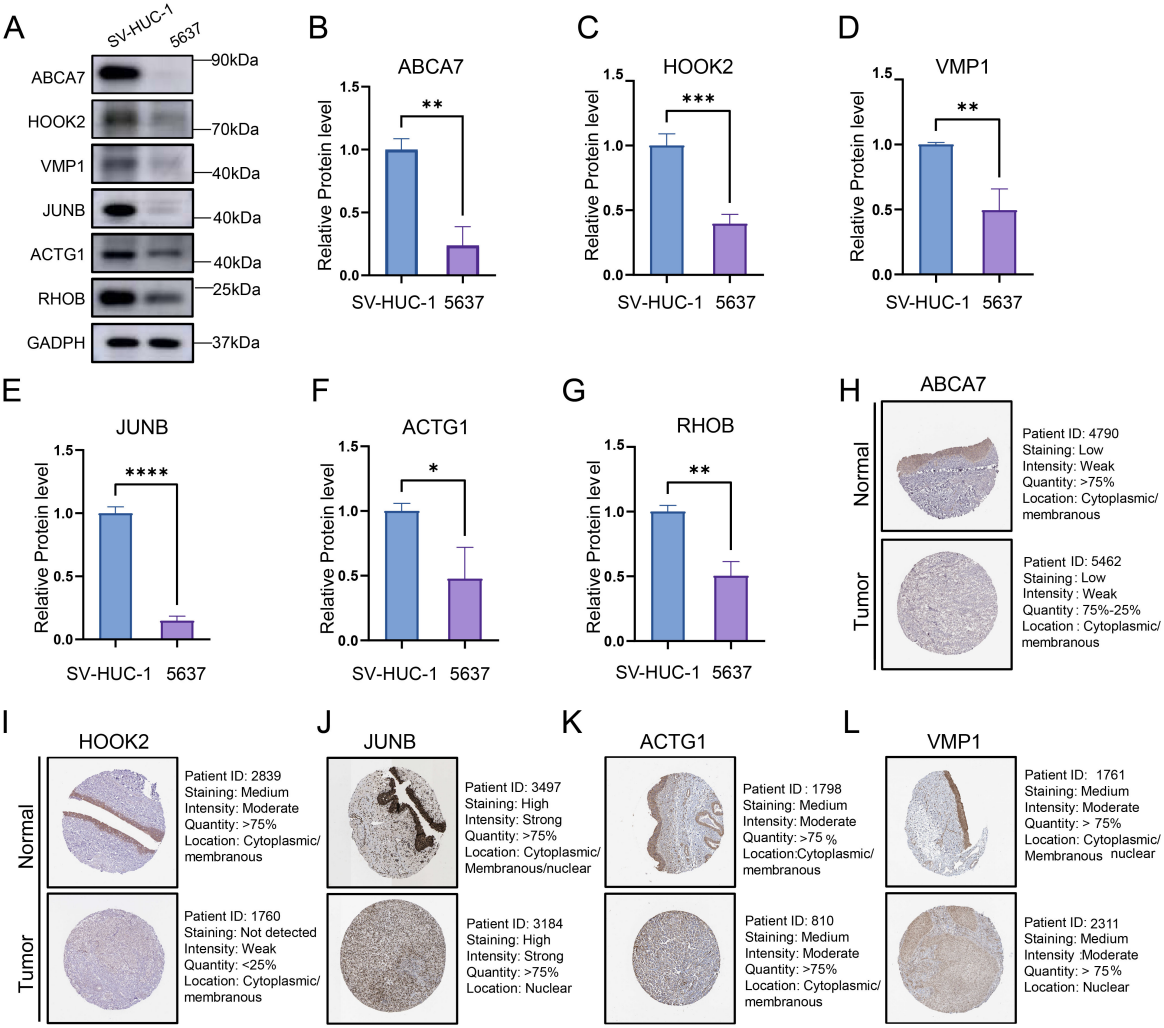
**(A)** Representative Western blot images showing the protein expression levels of six bladder cancer prognosis-associated genes. **(B-G)** Quantification of protein expression levels from three independent Western blot experiments. Data represent mean ± SD of triplicate measurements normalized to GAPDH. Statistical significance was determined by Student’s t-test, *P<0.05, **P<0.01, ***P<0.001, ****P<0.0001. **(H-L)** The protein expression levels of ABCA7, HOOK2, VMP1, JUNB and ACTG1 in normal tissues and bladder cancers from HPA database.

To investigate the protein expression profiles of six genes (ABCA7, HOOK2, JUNB, RHOB, VMP1, and ACTG1) that significantly impact both DSS and PFI in bladder cancer patients, we conducted Western blot analyses using SV-HUC-1 and 5637 cell lines. The results demonstrated significantly lower protein expression levels of these genes in bladder cancer cell lines compared to normal epithelial cell lines (**Figures 9A-G**). Subsequently, we further analyzed the expression patterns of these six genes in tumor tissue samples from patients using the HPA database. With the exception of RHOB, whose expression profile requires further clarification, the differential expression of the remaining proteins between bladder cancer and normal tissues was consistent with our analytical results (**Figures 9H-L**). Collectively, this study not only reveals the potential of these genes as novel pan-cancer therapeutic targets but also provides a theoretical foundation for precise prognostic stratification and biomarker-driven clinical management of BLCA patients.

## 4 Discussion

Resistance to tumor immunotherapy is a critical challenge hindering the beneficial treatment of cancer patients (2, 68). The heterogeneous response to immunotherapy across cancer types aggravates the need for biomarker research to optimize clinical selection and maximize survival benefits (69, 70). The high mutation frequency of EGFR in various human cancers inspired the hypothesis that EGFR could serve as a potential biomarker; however, the deterministic interaction relationship and mechanism between EGFR and TIME has not been fully reported (7, 71). Here, we utilized scRNA-Seq data of ICI cohorts to demonstrate the potential negative relationship between the EGFR scores of individual malignant cells and the response to immunotherapy. Subsequently, the interaction analysis of scRNA-Seq and bulk RNA-Seq in pan-cancer identified EGFR.Sig and confirmed this phenomenon. Notably, EGFR.Sig showed better predictive performance for immunotherapy response than previously published gene signatures. Based on multiple ML algorithms, we identified Hub-EGFR.Sig and found that it had an extensive relationship with the TIME and prognosis in pan-cancer. Focusing on BLCA, we further investigated the complex connection of Hub-EGFR.Sig and molecular typing, immune cell infiltration and drug sensitivity screening.

In this study, a generally negative relationship between EGFR scores and immunotherapy response was observed. This phenomenon that patients with most EGFR mutation have generally low response to ICIs is the most widespread concern in NSCLC (72–74). We excluded the interference of other cells in tumor tissues at the single-cell level and found that patients who responded to ICI treatment had lower EGFR scores. To further investigate the immune resistance of patients with high EGFR scores in pan-cancer, we developed EGFR.Sig based on the interactive analysis of 34 scRNA-Seq cohorts. The genes in EGFR.Sig are mainly enriched in pathways related to Rho GTPase effectors, which hold critical positions in multiple immune signal transducers serving as Ras homology (RHO) GTPases (75). Similar to other members of the small GTPase superfamily, Rho GTPase effectors have been reported to promote tumor immune evasion through MAPK- and β-catenin-related pathways (76). Therefore, we comprehensively screened the immune molecular landscape of EGFR.Sig across various cancers. As expected, results demonstrated that EGFR.Sig is positively correlated with most immune inhibitory genes and pathways but negatively correlated with antitumor immune cells in the TIME. Moreover, there is a strongly positive correlation between EGFR.Sig and ITH, which is consistent with previous studies (77, 78). For the commonly recognized ICI biomarker TMB, a previous explanation owed the lack of therapeutic effect in patients with EGFR mutations to its low expression (7, 79). However, failure to respond to ICIs still exists in many patients with a high TMB (80), which may lead to our observation of a positive correlation between EGFR.Sig and TMB. Altogether, the association between EGFR.Sig and tumor immunosuppression suggests its potential to serve as a predictive biomarker of immunotherapy.

Subsequently, multiple ML algorithms confirmed the capability of EGFR.Sig as a new predictive biomarker for ICI response. Compared with the previously published gene signatures, EGFR.Sig has better generalization and achieved an overall favorable performance in the independent verification set. In addition to these advantages, EGFR is widely recognized as one of the primary clinical targets for tumor therapy (7). The approval and ongoing clinical trials of various drugs have significantly enhanced the potential for future combined therapies to improve the response to immunotherapy. Moreover, the percentage of genes verified by *in vivo* and *in vitro* experiments in the CRISPR datasets proved the reliability of EGFR.Sig, in which MAT2A, JUNB, NR4A1 and C12orf57 were screened for combined strategies to overcome immune resistance. The increased expression of MAT2A (methionine adenosyltransferase 2A gene) in various cancers, including liver cancer, CRC, and gastric cancer, is recognized as a therapeutic target due to its role in regulating cell growth (81–84). Recent research has also found that the deletion of MAT2A mediated by CRISPR–Cas9 restricted the growth of hepatocellular carcinoma in mice by inhibiting T-cell exhaustion, which may be a potential strategy to enhance the ICI response of HCC (85). Moreover, JUNB (JunB proto-oncogene) and NR4A1 (nuclear receptor subfamily 4 group A member 1 gene) can also regulate the functional state of T cells in the TIME, serving as targets of tumor immunotherapy (86, 87). C12orf57 (chromosome 12 open reading frame 57) is found in the interactome atlas of receptor tyrosine kinase, although there are have been few more detailed studies to date (88). These studies suggest that the in-depth study of EGFR.Sig will contribute to the development of new strategies to strengthen tumor immunotherapy.

Furthermore, genes that are most critical to the immunotherapy response in EGFR.Sig were identified as Hub-EGFR.Sig. The above 4 genes screened by CRISPR are all in Hub-EGFR.Sig, and most of the other Hub-EGFR.Sig genes have also been reported to hold a position in the treatment of tumors. For example, RHOB (Ras homologue family member B gene) acts as a tumor suppressor most of the time, unlike other members of the Ras homologue family (89). RHOB can not only limit EGFR signal transduction on the cell surface (90) but also participate in antitumor immunity, such as antigen presentation and T-cell activation (89, 91). In our investigation, we also discovered that RHOB exhibited significant upregulation within the low-risk group of Hub-EGFR.Sig. Our Western blot analyses and HPA validation concordantly confirmed this expression pattern. In contrast, ABCA7 (ATP binding cassette subfamily A member 7) was expressed at a relatively higher level in the high-risk group. A recent study has shown that the inhibition of RHOB by hsa-miR-3178 can increase the expression of ABC transporter proteins through the PI3K/Akt pathway, which finally leads to the resistance of pancreatic cancer to gemcitabine (92). The antagonistic role of RHOB and ABCA7 in cancer reveals that there are complex interactions among Hub-EGFR.Sig, which was also confirmed in our subsequent miRNA–mRNA and TF-mRNA interactive network analysis. Notably, the Hub-EGFR.Sig risk scoring model integrates weighted contributions from multiple genes rather than relying on extreme expression changes of any single gene. This integrative approach more effectively captures the global biological characteristics of EGFR-related signaling and enhances the model’s robustness against technical variability and biological heterogeneity. Overall, Hub-EGFR.Sig encompasses a set of characteristic EGFR-related genes that play a vital role in determining the efficacy of immunotherapy, so it holds great potential as a novel and significant therapy target and prognostic biomarker.

In general, our study found that cancer patients with higher Hub-EGFR.Sig risk scores presented poorer DSS and PFI in pan-cancer. Elevated EGFR levels have a particularly strong association with the survival outlook of many cancers (93). Specific to individual cancer types, LUAD and LUSC have relatively high Hub-EGFR.Sig scores among cancers, and ample evidence has confirmed that EGFR tyrosine kinase inhibitors indeed prolong the PFS of NSCLC patients (94, 95). EGFR signal transduction also usually promotes the progression of BLCA (96, 97). However, the positive prognosis correlation of BLCA with Hub-EGFR.Sig observed in this study was not consistent with that in pan-cancer, although both DSS and PFI in BLCA showed the strongest association with Hub-EGFR.Sig. This observed heterogeneity primarily stems from differences in tumor microenvironment composition, molecular subtypes, MSI status, and variable responses to immunotherapy. Therefore, clinical interpretation of Hub-EGFR.Sig as a biomarker requires careful consideration of both the specific cancer type and underlying molecular context. Further investigation revealed that there were different correlations of Hub-EGFR.Sig with MSI across different cancer types. The relatively high Hub-EGFR.Sig score of BLCA was similar to that of LUAD and LUSC, while there was no significant correlation between Hub-EGFR.Sig and MSI in BLCA. MSI is observed at varied frequencies within malignant tumors, and its presence can serve as a predictor for cancer response to specific treatments, particularly immunotherapy (98, 99). Crosstalk between MSI affects the prognosis of tumors (100, 101), which may partly explain this phenomenon. Second, our study utilized DSS and PFI to better reflect the impact of immune therapy on the prognosis of BLCA (59). As we all know, BLCA is one of several tumors that responds positively to ICI treatment (102, 103). Our Hub-EGFR.Sig recognition by multiple ML algorithms was based on the importance of genes for immunotherapy, and deep analysis revealed that most of the genes in Hub-EGFR.Sig could independently affect the prognosis of BLCA. Thus, BLCA patients with higher Hub-EGFR.Sig scores may have better DSS and PFI in our study. Notably, our study was based on the transcriptional level analysis of EGFR-related genes. Tumor EGFR status can be evaluated by more than a dozen different methods, and the inconsistency in detection can lead to different results, which increases the variability between studies (93). In summary, Hub-EGFR.Sig can be used as a predictive biomarker for cancers despite the heterogeneity in various tumors.

Finally, we found that BLCA patients can be clustered into two subtypes according to Hub-EGFR.Sig, and there were some differences in the response of the two clusters to immunotherapy. Further comparing the infiltration of immune cells revealed an obvious difference in the TIME between the two clusters, which could be an important reason for the inconsistent response to immunotherapy. Although the continuous and complex crosstalk between multiple interrelated immune cells and tumors in BLCA still needs to be better characterized (104), several studies have confirmed that immune cell-related gene signatures can predict the clinical response of BLCA patients (105, 106). These findings reveal the possibility of targeted regulation of EGFR signaling combined with immunotherapy in the treatment of BLCA, which has rarely been reported in previous studies. By developing a new combination therapy, the response rate of immunotherapy can be improved (102, 107). The Hub-EGFR.Sig may serve as a valuable theoretical foundation for screening potential novel therapeutic targets, developing precision combination therapies, and formulating personalized treatment regimens. Therefore, we further screened the potentially available drugs for tumor immune regulation based on Hub-EGFR.Sig. These drugs included antineoplastic drugs such as etoposide phosphate, sparsomycin and vincristine, which will contribute to the design of new combined cancer treatment strategies.

Certainly, our research has several limitations. First, this study primarily depended on transcriptomic data, whether scRNA-Seq or bulk RNA-Seq, which may have technically heterogeneous. To investigate the relationship between EGFR and cancer cell immunotherapy response, we included two scRNA-Seq ICI cohorts with clear clinical outcomes from the GEO database. We noted that these two cohorts differed in their single-cell building and sequencing processes, resulting in different numbers of patients and cells in each group. In response to possible technical heterogeneity, stringent quality control measures were taken, including the exclusion of low-quality cells and normalization of the different datasets. Batch effect correction was performed before downstream analyses. In addition, screening of EGFR.Sig genes was based on the integration and cross-validation of multiple cohorts and multiple ML algorithms, thereby minimizing the impact of technical bias in any single dataset. This approach ensured the robustness and generalization ability of the identified EGFR-related signature genes. Moreover, the selection of data and the lack of information on the retrospective pan-cancer cohorts may have a potential impact on our results. We employed well-established normalization and batch correction approaches, including the Harmony package for single-cell data and the COMPAT tool for bulk RNA-Seq datasets, to ensure the reliability of downstream analyses. While these methods effectively address major technical variations, we acknowledge that certain potential biases may require development of more sophisticated statistical approaches for comprehensive detection in future studies. Finally, although multiple ML algorithms were used and many published cohorts were included to develop and verify EGFR.Sig, it will be necessary to verify this signature in future experiments and clinical validation studies.

## 5 Conclusion

In conclusion, our research offers novel insights into EGFR linked to immunotherapy response and prognosis in pan-cancer. Using multiple ML algorithms based on interactive analysis of scRNA-Seq and bulk RNA-Seq data, we successfully devised an improved predictive biomarker for immunotherapy and identified Hub-EGFR.Sig to explore the heterogeneity of different cancer prognoses. Focusing on BLCA, we investigated its interaction with molecular typing, TIME and drug sensitivity screening. The present study provides a potential pan-cancer targeting strategy related to clinical treatment for precision oncology, which will help to improve tumor immunotherapy and benefit patients.

## Data Availability

The original contributions presented in the study are included in the article/**Supplementary Material**. Further inquiries can be directed to the corresponding authors.
