# Supplementary material for: Comprehensive analysis of single-cell and bulk RNA sequencing data reveals an EGFR signature for predicting immunotherapy response and prognosis in pan-cancer
Source: Front Immunol. 2025 Jun 12;16:1604394. doi: 10.3389/fimmu.2025.1604394 (PMC12198250; doi:10.3389/fimmu.2025.1604394)
Supplement: Supplementary file 1 [file DataSheet1.zip › Supplementary figures S1-S5.DOCX]

**Comprehensive analysis of single-cell and bulk RNA sequencing data reveals an EGFR signature for predicting immunotherapy response and prognosis in pan-cancer**

**Supplementary figures**


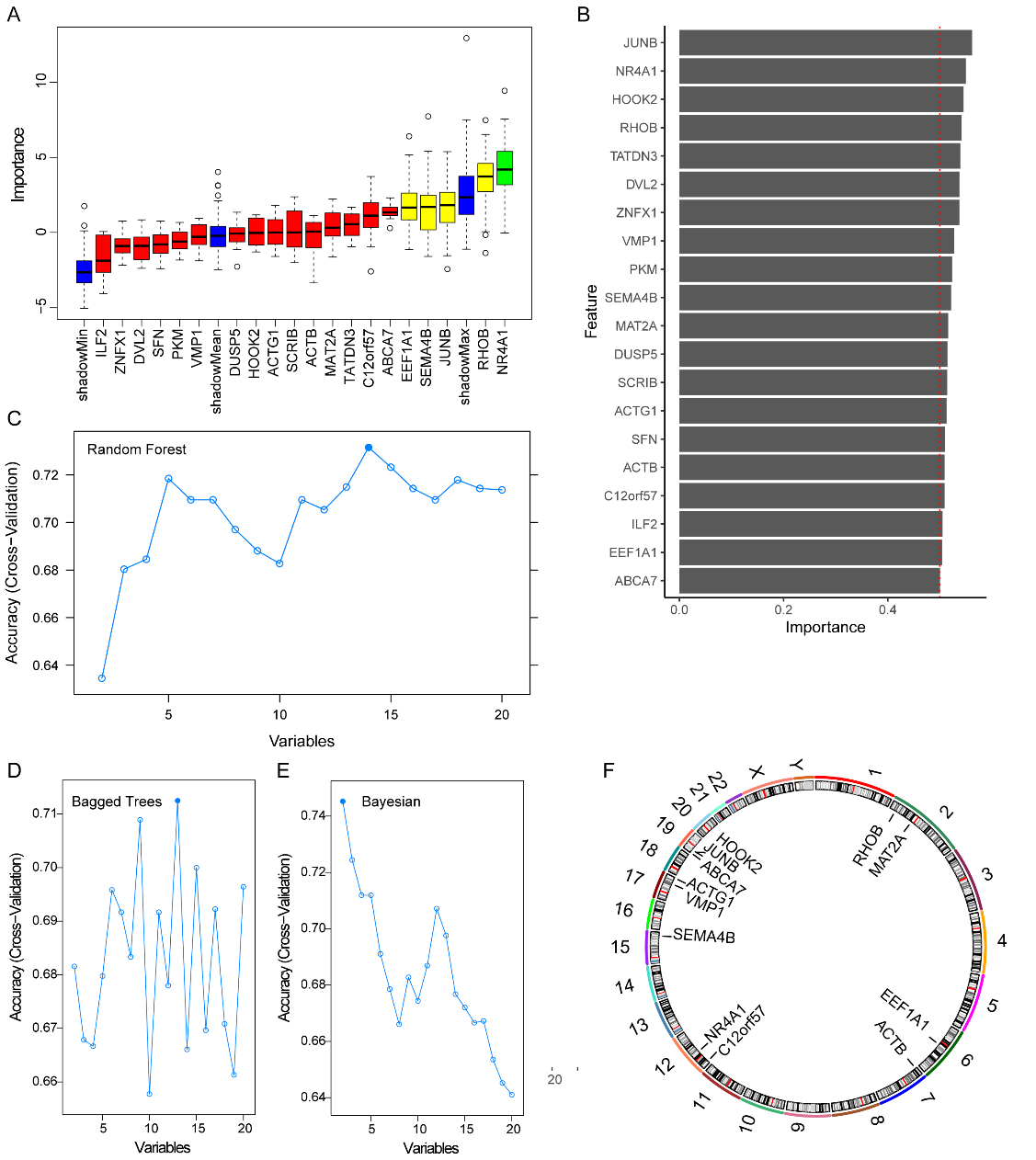


**Figure S1. Screening of the Hub-EGFR.Sig.** A. Diagnostic model of Wrapper. B. Diagnostic model of Learning Vector Quantization. C. Diagnostic model of Random Forest. D. Diagnostic model of Bagged Trees. E. Diagnostic model of Bayesian. F. Chromosome mapping of Hub-EGFR.Sig.


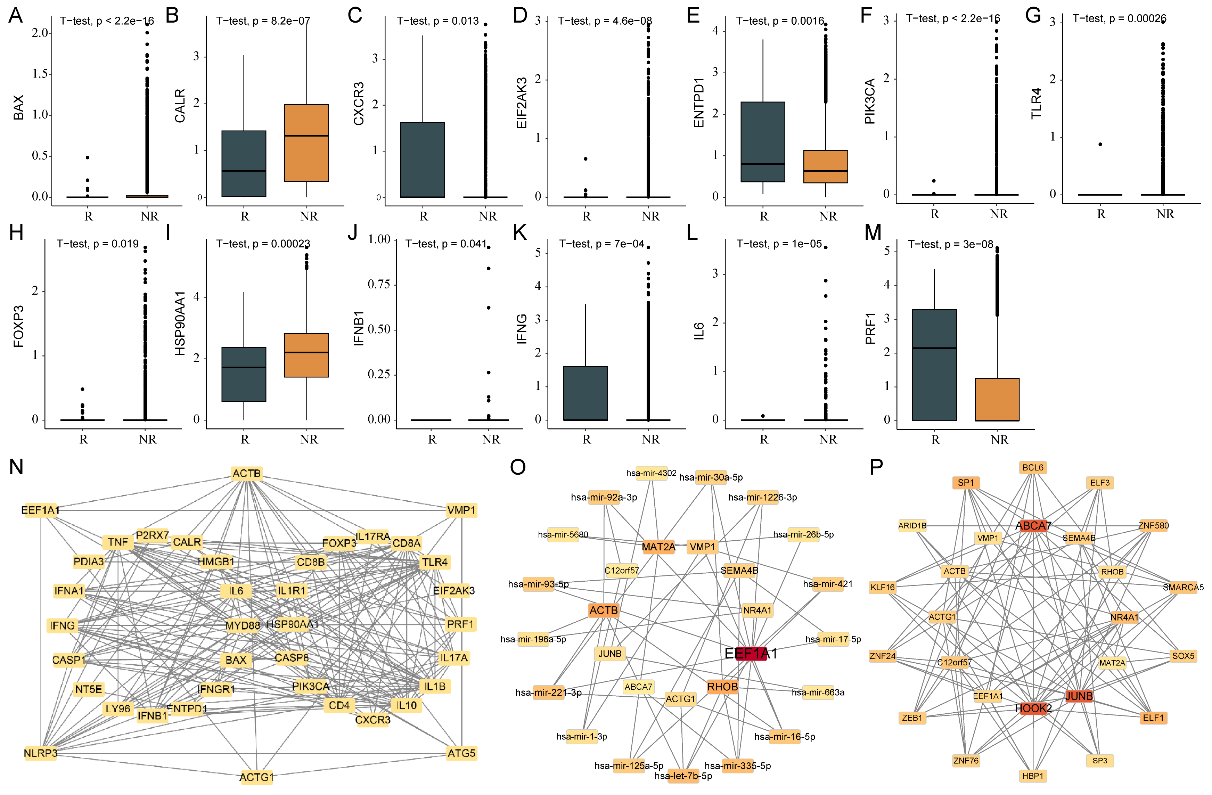


**Figure S2. Protein interactions of genes in Hub-EGFR.Sig.** A-M. Expression of ICD related genes in different immunotherapy response groups. N. Protein-protein interaction network of ICD related genes and Hub-EGFR.Sig. O. The mRNA-miRNA regulatory network of Hub-EGFR.Sig. P. TF-mRNA regulatory network of Hub-EGFR.Sig. The darker color indicates the richer interaction. Nodes without interaction have been removed.


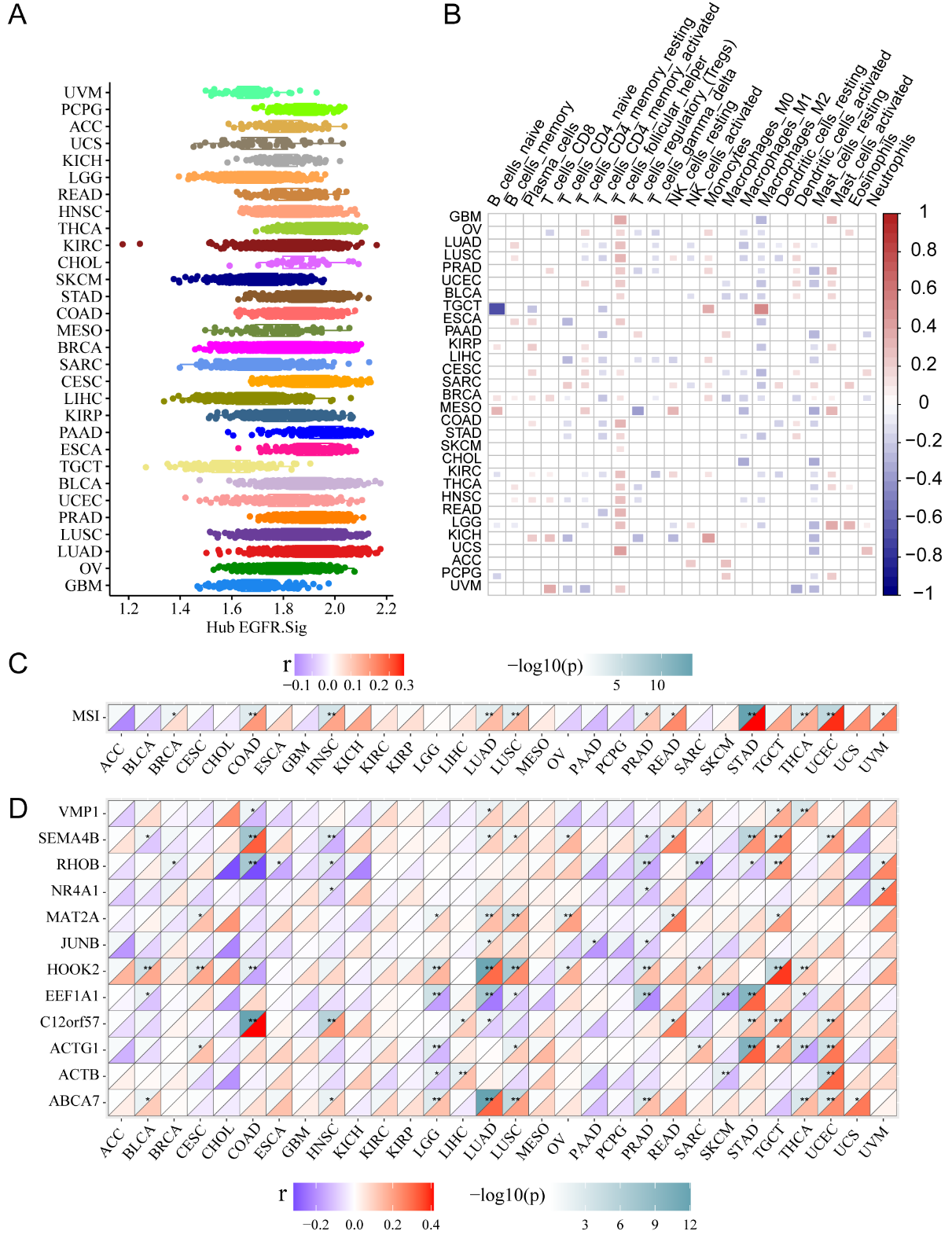


**Figure S3. Landscape of Hub-EGFR.Sig in pan-cancer.** A. The expression of Hub-EGFR.Sig in different cancers. B. The correlation between the Hub-EGFR.Sig and immune cell infiltration in different cancers. Red represents positive correlation, blue represents negative correlation and the darker the color, the stronger the correlation. C. The correlation between Hub-EGFR.Sig and MSI in different cancers. D. The correlation between the expression levels of 12 genes in Hub-EGFR.Sig and MSI. Color depth represents the value of correlation coefficient r, and **p*<0.05, ***p*<0.01.


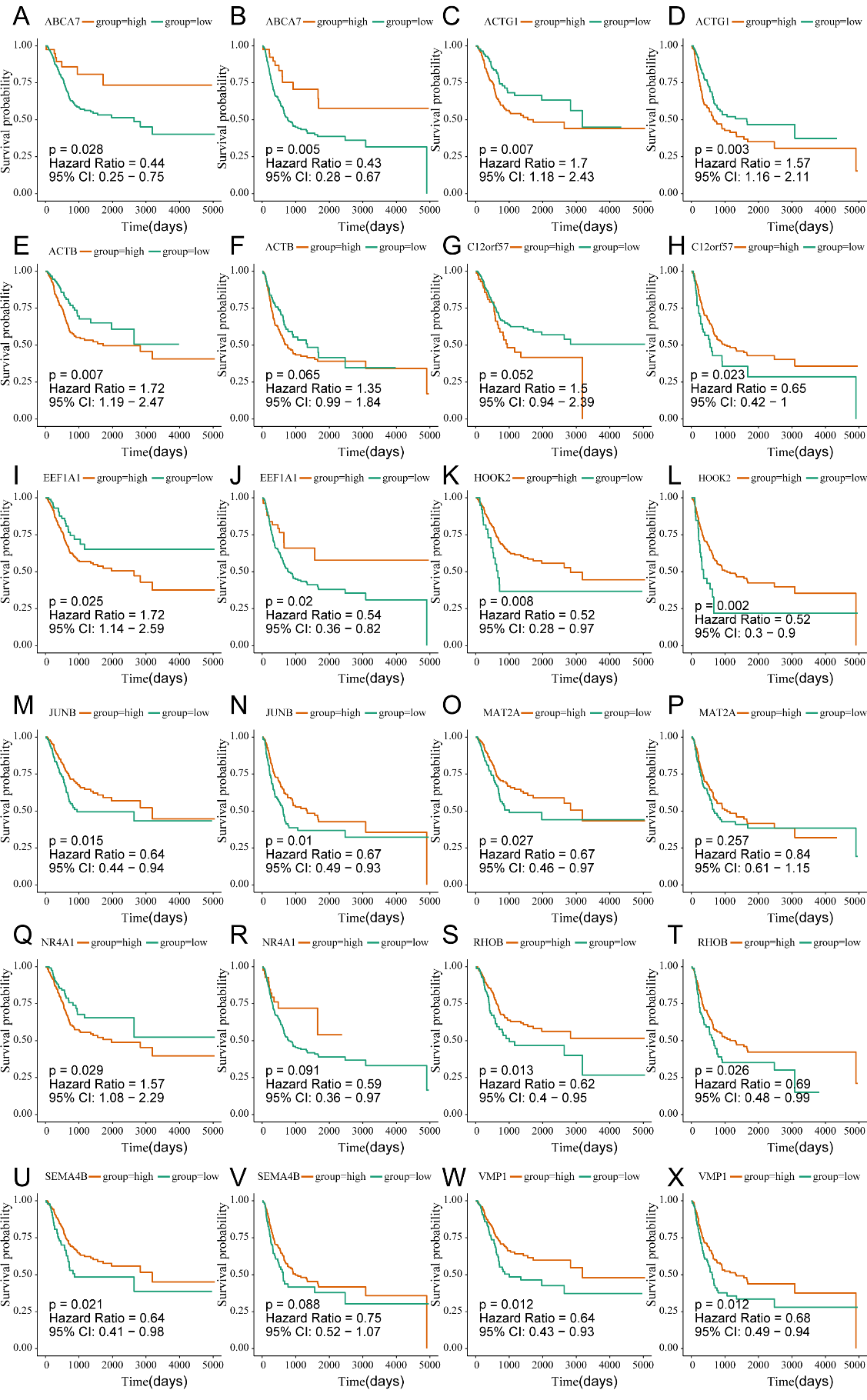


**Figure S4. DSS and PFI between the high and low expression groups of Hub-EGFR.Sig in bladder cancer.** For each gene, the K-M curve on the left shows DSS, and the K-M curve on the right shows PFI.


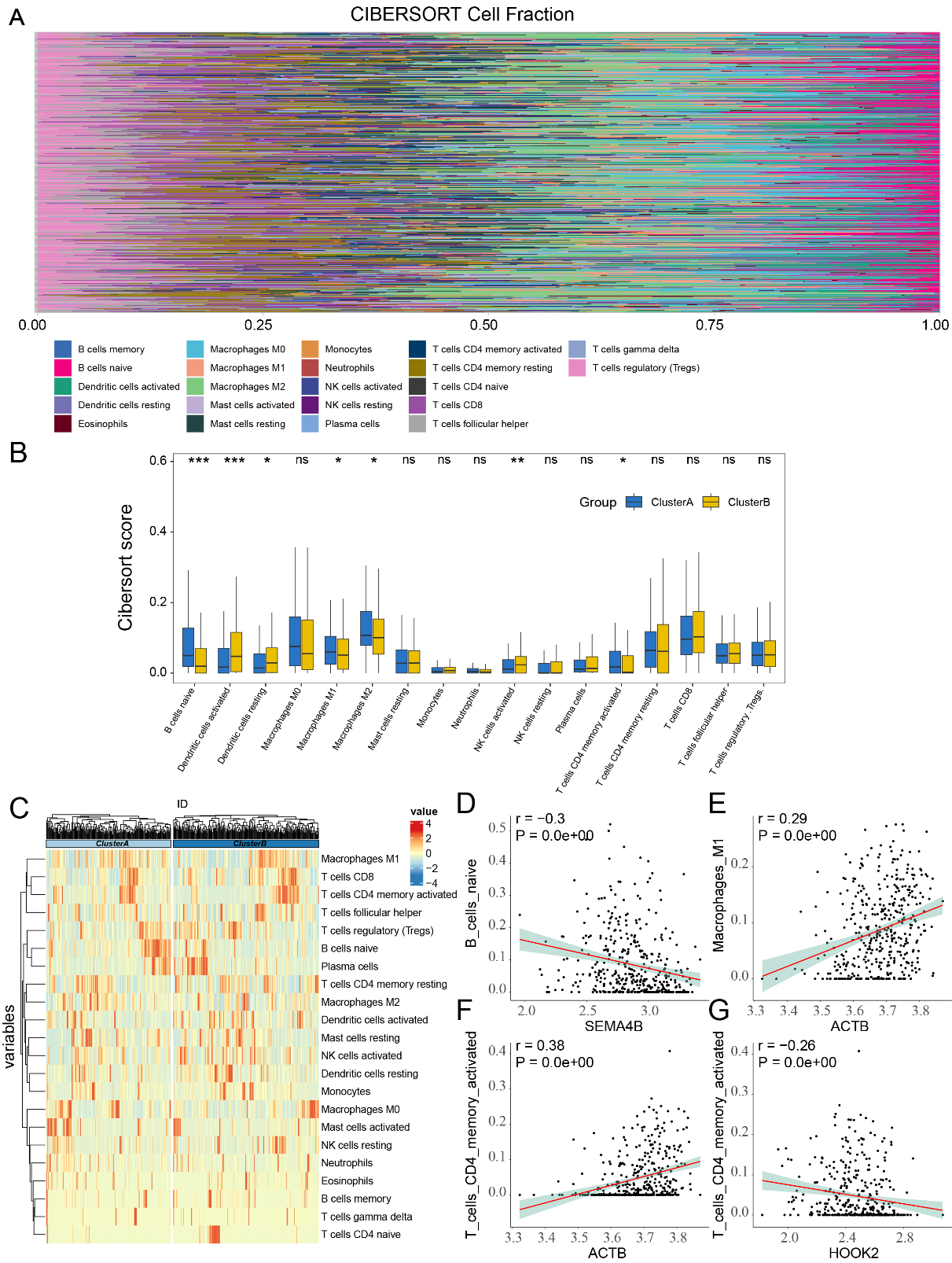


**Figure S5. Immune cell infiltration in BLCA of different Hub-EGFR.Sig subtypes (Cibersort).** A. The proportion of immune cells in the TIME of BLCA. B. Comparing the abundance of immune cell infiltration in BLCA Hub-EGFR.Sig subtypes. **p*<0.05, ***p*<0.01, ****p*<0.001 and ns means no significant. C. Heatmap of immune cell infiltration abundance in the two Hub-EGFR.Sig subtypes of BLCA. D-G. Correlation analysis of genes in Hub-EGFR.Sig and immune cell infiltration abundance in BLCA.
