# Supplementary material for: Comprehensive analysis of single-cell and bulk RNA sequencing data reveals an EGFR signature for predicting immunotherapy response and prognosis in pan-cancer
Source: Front Immunol. 2025 Jun 12;16:1604394. doi: 10.3389/fimmu.2025.1604394 (PMC12198250; doi:10.3389/fimmu.2025.1604394)
Supplement: Supplementary file 1 [file DataSheet1.zip › Supplementary tables S1-S4.DOCX]

**Comprehensive analysis of single-cell and bulk RNA sequencing data reveals an EGFR signature for predicting immunotherapy response and prognosis in pan-cancer**

**Supplementary tables**

**Table S1. EGFR-related genes obtained from the Gene Set Enrichment Analysis database.**

| ABCA7 | ACTB | ACTG1 | C11orf98 | C12orf57 |
| --- | --- | --- | --- | --- |
| DUSP5 | DUSP5-DT | DVL2 | EEF1A1 | H2BC18 |
| HOOK2 | ILF2 | JUNB | LINC02709 | MALAT1 |
| MIR4530 | NR4A1 | PKM | RHOB | RNU7-1 |
| RNVU1-27 | RPL36AP44 | SCRIB | SEMA4B | SFN |
| SNORD12C | TATDN3 | TM4SF1-AS1 | VMP1 | ZFAS1 |

**Table S2. List of pan-cancer signatures for immunotherapy.**

| **Signatures** | **Algorithms** | **Description** | **Gene lists** |
| --- | --- | --- | --- |
| T.cell.inflamed.Sig^[1]^ | Average gene expression | An IFN-gamma-related profile that predicts PD-1blockade efficacy | CD3D, IDO1, CIITA, CD3E, CCL5, GZMK, CD2, HLA-DRA, CXCL13, IL2RG, NKG7, HLA-E, CXCR6, LAG3, TAGAP, CXCL10, STAT1, GZMB |
| IFNG.Sig^[1]^ | Average gene expression | T-cell inflamed gene expression profiles | IFNG, STAT1, IDO1, CXCL10, CXCL9, HLA-DRA |
| PD-L1.Sig^[2]^ | Expression of PD-L1 | Gene expression profiles of PD-L1 / PDCD1. | PDL1, PDCD1 |
| Cytotoxic.Sig^[3]^ | Geometric mean of gene expression | Genes associated with cytotoxic activities | GZMA, PRF1 |

**Table S3. Gene list of EGFR.Sig.**

| ABCA7 | ACTB | ACTG1 | C12orf57 | DUSP5 |
| --- | --- | --- | --- | --- |
| DVL2 | EEF1A1 | HOOK2 | ILF2 | JUNB |
| MAT2A | NR4A1 | PKM | RHOB | SCRIB |
| SEMA4B | SFN | TATDN3 | VMP1 | ZNFX1 |

**Table S4. Immune-related genes previously published in the pan-cancer TCGA cohort^[4]^.**

| ADORA2A | ARG1 | BTLA | BTN3A1 | BTN3A2 | C10orf54 |
| --- | --- | --- | --- | --- | --- |
| CCL5 | CD27 | CD274 | CD276 | CD28 | CD40 |
| CD40LG | CD70 | CD80 | CTLA4 | CX3CL1 | CXCL10 |
| CXCL9 | EDNRB | ENTPD1 | GZMA | HAVCR2 | HLA-A |
| HLA-B | HLA-C | HLA-DPA1 | HLA-DPB1 | HLA-DQA1 | HLA-DQA2 |
| HLA-DQB1 | HLA-DQB2 | HLA-DRA | HLA-DRB1 | HLA-DRB5 | HMGB1 |
| ICAM1 | ICOS | ICOSLG | IDO1 | IFNA1 | IFNA2 |
| IFNG | IL10 | IL12A | IL13 | IL1A | IL1B |
| IL2 | IL2RA | IL4 | ITGB2 | KIR2DL1 | KIR2DL3 |
| LAG3 | MICA | MICB | PDCD1 | PDCD1LG2 | PRF1 |
| SELP | SLAMF7 | TGFB1 | TIGIT | TLR4 | TNF |
| TNFRSF14 | TNFRSF18 | TNFRSF4 | TNFRSF9 | TNFSF4 | TNFSF9 |
| VEGFA | VEGFB | VTCN1 |  |  |  |

Reference

1. Ayers M, Lunceford J, Nebozhyn M, Murphy E, Loboda A, Kaufman DR, et al. IFN-γ-related mRNA profile predicts clinical response to PD-1 blockade. J Clin Invest. 2017;127(8):2930-40.

2. Topalian SL, Hodi FS, Brahmer JR, Gettinger SN, Smith DC, McDermott DF, et al. Safety, activity, and immune correlates of anti-PD-1 antibody in cancer. N Engl J Med. 2012;366(26):2443-54.

3. Rooney MS, Shukla SA, Wu CJ, Getz G, Hacohen N. Molecular and genetic properties of tumors associated with local immune cytolytic activity. Cell. 2015;160(1-2):48-61.

4. Thorsson V, Gibbs DL, Brown SD, Wolf D, Bortone DS, Ou Yang TH, et al. The Immune Landscape of Cancer. Immunity. 2018;48(4):812-30.e14.
